# All-optical interrogation of neural circuits in behaving mice

**DOI:** 10.1038/s41596-022-00691-w

**Published:** 2022-04-27

**Authors:** Lloyd E. Russell, Henry W. P. Dalgleish, Rebecca Nutbrown, Oliver M. Gauld, Dustin Herrmann, Mehmet Fişek, Adam M. Packer, Michael Häusser

**Affiliations:** 1 Wolfson Institute for Biomedical Research, University College London, London, UK; 2 Sainsbury Wellcome Centre, University College London, London, UK; 3 Department of Physiology, Anatomy and Genetics, University of Oxford, Oxford, UK

## Abstract

Recent advances combining two-photon calcium imaging and two-photon optogenetics with computer-generated holography now allow us to read and write the activity of large populations of neurons in vivo at cellular resolution and with high temporal resolution. Such ‘all-optical’ techniques enable experimenters to probe the effects of functionally defined neurons on neural circuit function and behavioral output with new levels of precision. This greatly increases flexibility, resolution, targeting specificity and throughput compared with alternative approaches based on electrophysiology and/or one-photon optogenetics and can interrogate larger and more densely labeled populations of neurons than current voltage imaging-based implementations. This protocol describes the experimental workflow for all-optical interrogation experiments in awake, behaving head-fixed mice. We describe modular procedures for the setup and calibration of an all-optical system (~3 h), the preparation of an indicator and opsin-expressing and task-performing animal (~3–6 weeks), the characterization of functional and photostimulation responses (~2 h per field of view) and the design and implementation of an all-optical experiment (achievable within the timescale of a normal behavioral experiment; ~3–5 h per field of view). We discuss optimizations for efficiently selecting and targeting neuronal ensembles for photostimulation sequences, as well as generating photostimulation response maps from the imaging data that can be used to examine the impact of photostimulation on the local circuit. We demonstrate the utility of this strategy in three brain areas by using different experimental setups. This approach can in principle be adapted to any brain area to probe functional connectivity in neural circuits and investigate the relationship between neural circuit activity and behavior.

## Introduction

**Fig. 1:**
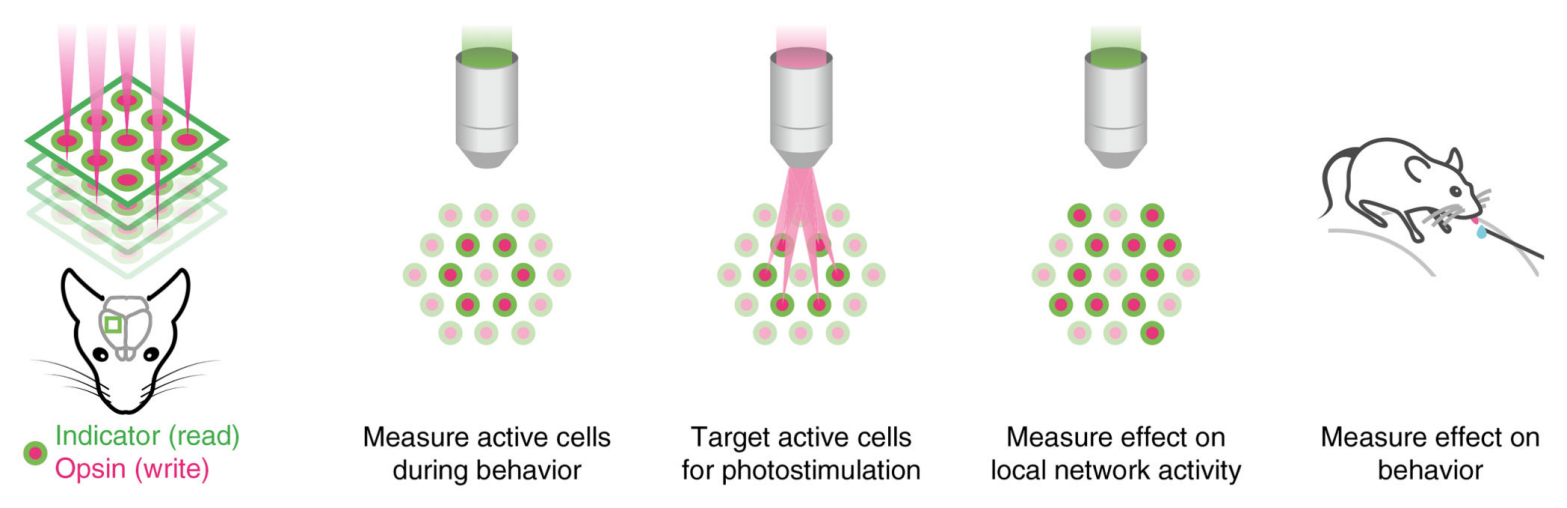
Conceptual goals of all-optical interrogation experiments. Schematic diagram illustrating the basic elements of all-optical interrogation studies, showing the typical sequence used in an experiment. Indicators are used to read neural activity (both to identify cells active during a behavior/sensory manipulation and to measure any influence of all-optical interrogation on the local network; second and fourth images from the left), and opsins are used to write neural activity (middle image).

A fundamental goal in neuroscience is to understand how the brain encodes information in patterns of neural activity that can be used to guide behavior. Addressing this challenge requires methods that allow for the controlled manipulation of neuronal activity in vivo to determine which features of neural activity are most relevant to the behavior^1,2^, such as the spike rate^3,4^, spike timing^5–8^, spike number^7,9^ and the functional identity^10,11^ and spatial distribution of the neurons active during the behavior. These questions can now start to be addressed by using a recently introduced ‘all-optical’ experimental strategy^12–21^, which leverages the proliferation of light-based tools in neuroscience to combine two-photon calcium imaging^22–26^ for reading neural activity with two-photon optogenetics^27–30^ and computer-generated holography^31,32^ or generalized phase contrast^33^ for writing it, with a view to addressing the limitations of existing electrophysiological and/or one-photon optogenetic approaches^34^. This approach thus allows flexible, simultaneous reading and writing of neural activity in vivo (Fig. 1) and has been made possible by the combined effort of many laboratories that have developed elements of the all-optical toolkit and/or combined them to achieve successful implementations of the strategy^13–17,19–21,35–39^. This approach has already been used for a wide range of experiments, including mapping functional connectivity of circuits^19,20,37,40–42^ and modulation of behavior through the targeted manipulation of functionally defined neurons in several brain areas^18,20,21,37,40–44^. As all-optical interrogation becomes more widely used, it is crucial that the potential pitfalls and limitations of the approach are recognized and that rigorous standards are set by the field to facilitate the implementation and interpretation of experiments using this approach.

The all-optical approach is challenging, regardless of the specific implementation, because it requires many complex experimental procedures, as well as coordinated interaction between multiple software and hardware modules. For example, all-optical systems involve two light paths, one for imaging and one for photostimulation. These light paths each comprise laser sources, power modulation devices, beam-steering mirrors and, on the photostimulation side, beam-patterning devices to enable multiple neurons to be stimulated at once. The imaging light path may use volumetric scanning with various methods (electrically tunable lenses, piezo elements or spatial light modulators) to enable larger populations to be recorded and thus targeted by the photostimulation pathway^16,37,41,45^. These two light paths must be calibrated such that they are co-registered, allowing the photostimulation laser to hit precise locations in the imaging field of view (FOV). To perform experiments, the photostimulation laser is targeted toward neurons that have typically been identified by some anatomical or functional property. These targeted neurons must of course co-express two proteins, an indicator and an effector, enabling the cell’s activity to be read out as well as manipulated. Finally, the entire system should be a closed loop, such that the stimulation of neurons can be triggered either by monitored neural activity patterns or by an external event, such as behavioral state or task.

### Overview of the protocol

**Fig. 2:**
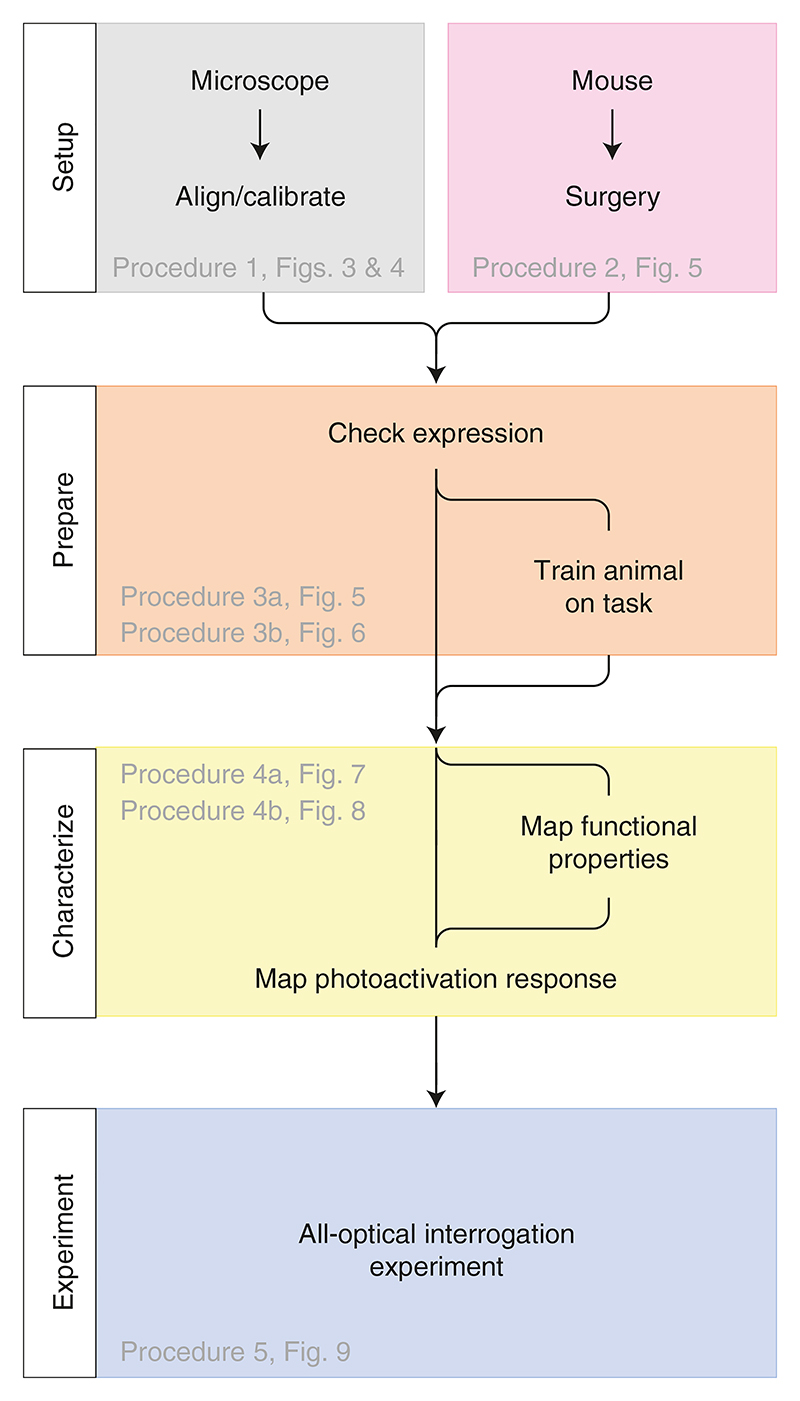
Overview of experimental steps. Essential steps, common to all all-optical experiments. The microscope must be aligned and calibrated before use. Animals used for experiments are engineered to express an activity indicator and opsin in specific neuronal populations. The expression of these constructs is assessed. Animals are (optionally) trained on a behavioral task. Neural responses to a stimulus/task variable of interest are mapped. Neural responses to photostimulation are mapped to identify photostimulatable cells. Finally, an experiment can be performed whereby functionally characterized neurons are targeted for photostimulation during a behavior of interest.

**Fig. 3:**
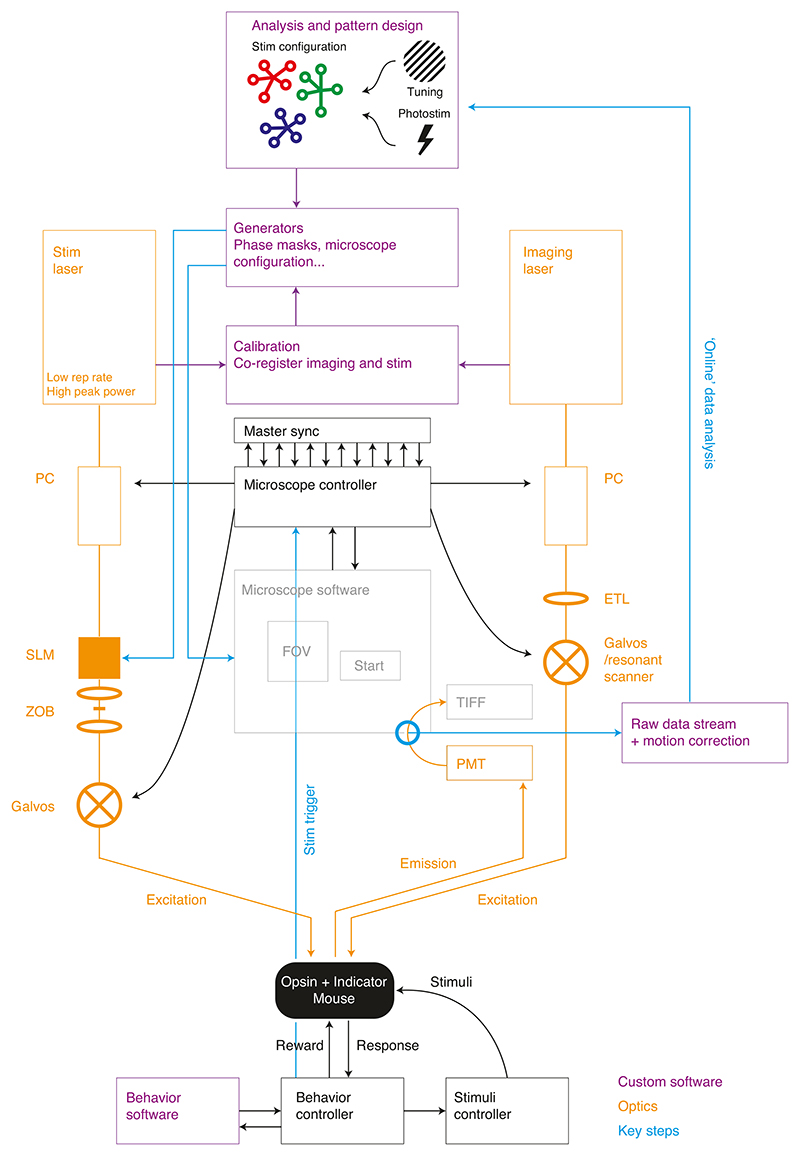
System diagram. To perform all-optical experiments, custom software is used to generate stimulation patterns targeted to neurons of interest as identified by analysis of imaging data. The stimulation patterns are generated in the form of files to load into different microscope software modules interfacing with the optical components and require the use of predetermined calibrations. The stimulation pattern files configure the system such that external triggers (e.g., from a behavioral experiment) can trigger the stimulation of particular neurons by determining the diffraction pattern caused by the SLM and driving power-modulation devices as well as galvanometer mirrors. ETL, electrically tunable lens; Galvos, galvanometers; PC, computer; PMT, photomultiplier tube; Photostim, photostimulation; rep, repetition; SLM, spatial light modulator; Stim, stimulation; sync, synchronization; ZOB, zero-order block.

**Fig. 4:**
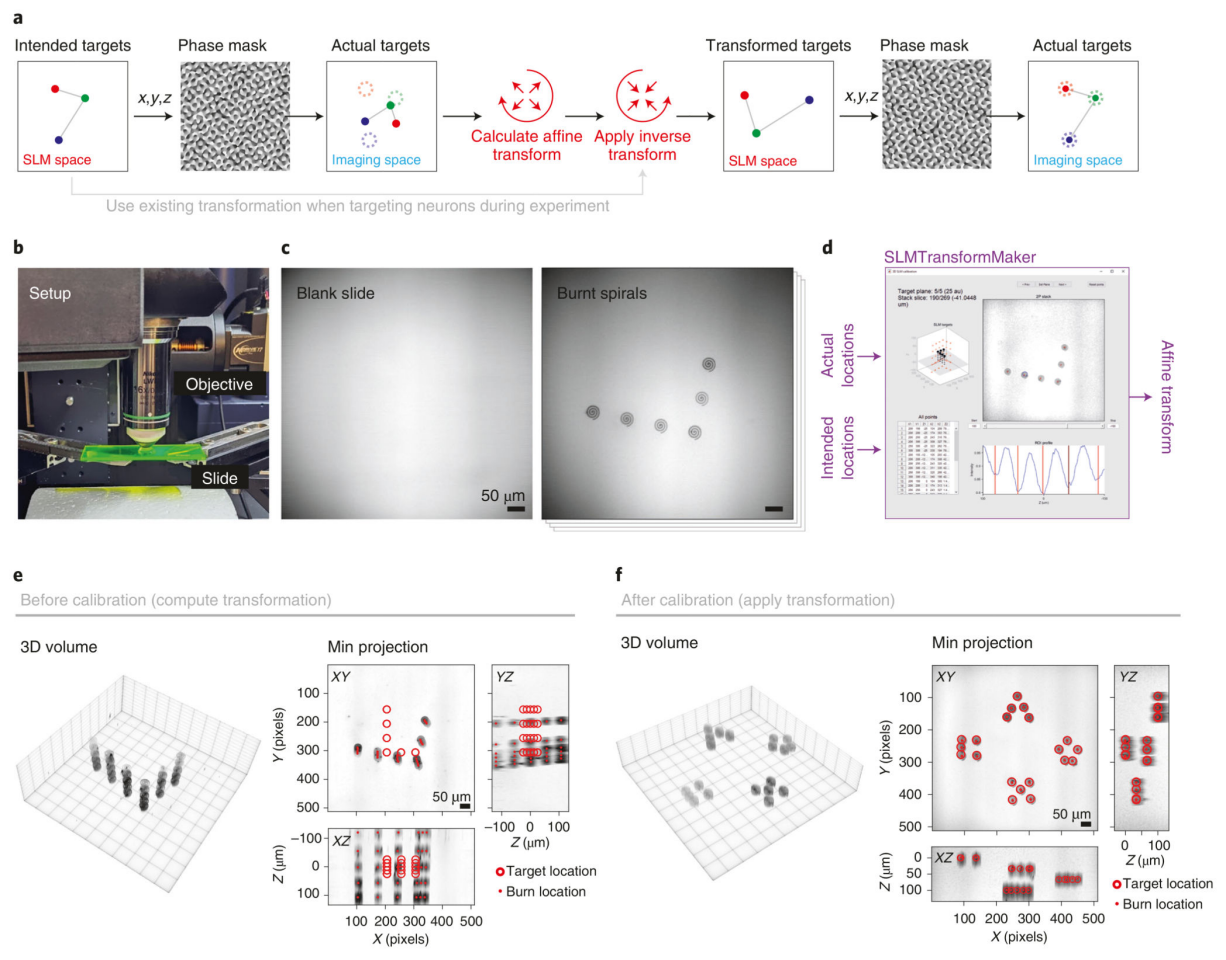
SLM calibration: mapping photostimulation targets to imaging coordinates. **a**, Without calibrating the SLM coordinates, the intended coordinates converted into the diffraction pattern generated by the SLM (via its displayed phase mask) will focus at arbitrary locations of the imaging FOV, precluding accurate targeting of precise neurons. By mapping the transformation between programmed SLM coordinates and ultimate location on the imaging FOV (usually by a collinearity-preserving affine transformation), the inverse can be applied, allowing for precise targeting of neurons. **b**, Photograph of a fluorescent plastic slide, used for calibration, imaged by the microscope objective. **c**, Images of the fluorescent slide acquired by the imaging pathway (left) and the same slide after programming the SLM and burning the resulting spots into the slide by spiral scanning (right). **d**, Software (SLMTransformMaker) is used to compute the transformation between SLM target coordinates and the imaging FOV locations of those SLM spots after burning them into a plastic slide. **e**, 3D projection of a volumetric stack taken of the burnt SLM spots (burnt on five axial planes) acquired for the calibration process. **f**, 3D projection of a different set of SLM patterns, but after calibration, demonstrating successful targeting to intended locations. Min projection = minimum intensity projection.

**Fig. 5:**
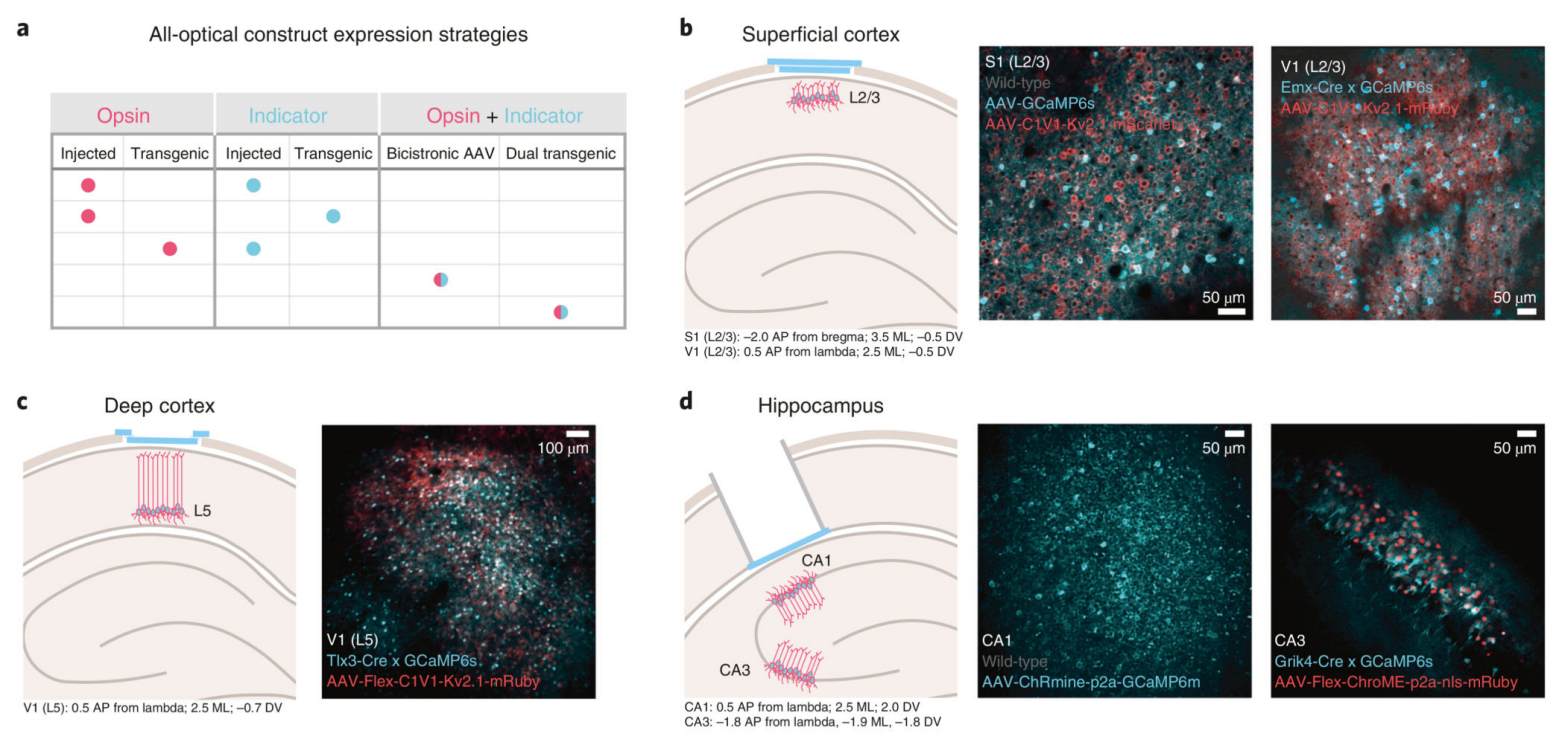
Inducing and checking expression of all-optical constructs. **a**, Strategies for achieving co-expression of all-optical constructs. **b**, Co-expression of all-optical constructs in superficial primary somatosensory and visual cortices (L2/3 S1 and V1). Left, experimental prep schematic; a chronic imaging window is installed on the cortical surface with either dual adeno-associated virus (AAV) expression of GCaMP and C1V1 (S1) or AAV expression of C1V1 in GCaMP transgenic mice (V1). Two right-hand images, example of healthy co-expression. **c**, Co-expression of all-optical constructs in deep cortex (L5 V1). Left, experimental preparation schematic; a chronic imaging window is installed on the cortical surface of L5-Cre transgenic mice injected with FLEX-C1V1 and FLEX-GCaMP. Right, example of healthy co-expression. **d**, Co-expression of all-optical constructs in subcortical structures (hippocampal CA1 and CA3). Left, experimental preparation schematic; cortical aspiration combined with a canula + chronic imaging window in CA1/CA3-Cre transgenic mice injected with FLEX-C1V1 and FLEX-GCaMP. Two right-hand images, example of healthy co-expression. AP, antero-posterior; bregma, junction point of the coronal and sagittal sutures of the skull; DV, dorso-ventral; lambda, junction point of the lambdoid and sagittal sutures of the skull; ML, medio-lateral.

**Fig. 6:**
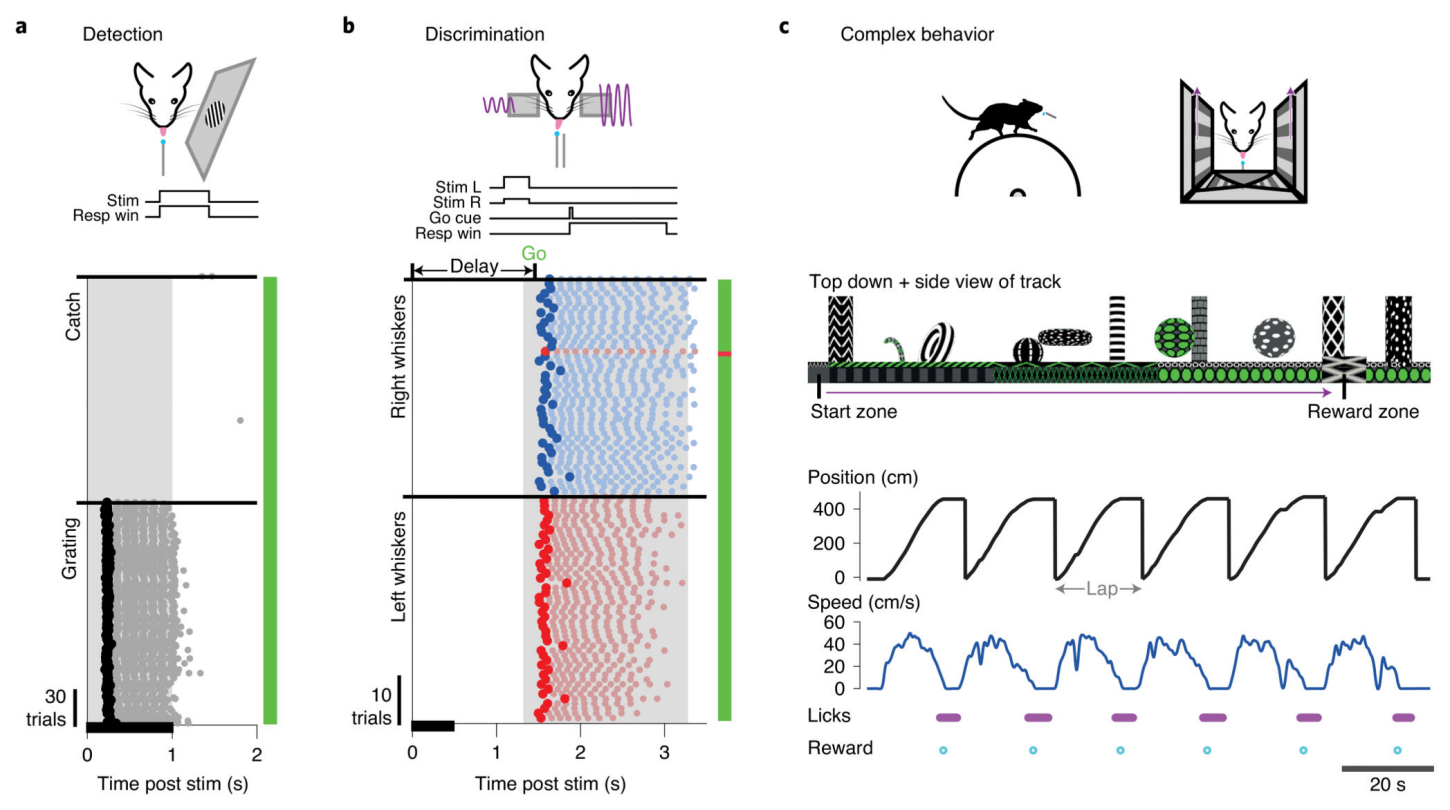
Choosing an appropriate behavioral paradigm. **a**, Example of a visual detection behavior. Top, task schematic; mice are required to report the presence of a randomly oriented drifting grating on a monitor by licking for a sucrose reward at an electronic lickometer. Bottom, sorted lick raster vertically stacking poststimulus epochs indicating the stimulus duration (black horizontal bar along x-axis), first lick on each trial (black dot) and subsequent trial licks (gray dots). Task performance is indicated by the color bar on the right, where color at each vertical position indicates performance on the corresponding trial in the trial raster to the left. Green, correct response (lick on grating trials; withhold on catch trials); red, incorrect response (lick on catch trials); gray, miss (no lick on grating trials). Note that because this animal is well trained, all trials are correct (green). Note also that trials were delivered pseudorandomly but sorted for display. *N* = 1 mouse, 1 training session, 367 trials. **b**, Example of a delay whisker discrimination behavior. Top, task schematic; mice are required to report which whisker pad receives the highest amplitude sinusoidal piezo vibration of two simultaneously delivered bilateral whisker stimuli by licking for sucrose rewards at one of two lickometer ports after an auditory tone Go cue signaling the end of a 1.5-s delay period after stimulus onset. Bottom, sorted lick raster vertically stacking poststimulus epochs; the conventions are the same as in **a** except that right and left licks are colored red and blue, respectively. As in **a**, task performance is indicated by the color bar on the right with slightly different color conventions. Green, correct response (lick left on left whisker stimulation trials; lick right on right whisker stimulation trials); red, incorrect response (lick right on left whisker stimulation trials; lick left on right whisker stimulation trials); gray, miss (no lick on either trial type). Note that because this animal is well trained, almost all trials are correct for both contingencies (green). Note also that trials were delivered pseudorandomly but sorted for display. *N* = 1 mouse, 1 training session, 101 trials. **c**, Example of a complex spatial navigation paradigm. Top, task schematic; mice are head-fixed on a cylindrical treadmill that controls movement through a virtual linear world displayed on three surrounding monitors. They spawn at the start of the virtual track and are required to run the length of the track before stopping and licking in the designated reward zone. Middle, virtual linear track indicating task features, start zone and reward zone. Note that the track running continuously left to right is a top-down view, whereas landmarks/objects rising from this are side view. Bottom, position along track, speed, lick times and reward deliveries for successive laps along the track. *N* = 1 mouse, 1 training session, 6 laps along track. Go cue, go cue tone epoch; Catch, catch trials (no stimulus/cue delivered); Grating, stimulus trials on which a visual grating was displayed; Left whiskers, trials on which left whiskers were stimulated; Resp win, behavioral response (lick) window epoch; Right whiskers, trials on which right whiskers were stimulated; Stim, stimulus epoch; Stim L, left whisker stimulus epoch and amplitude; Stim R, right whisker stimulus epoch and amplitude.

**Fig. 7:**
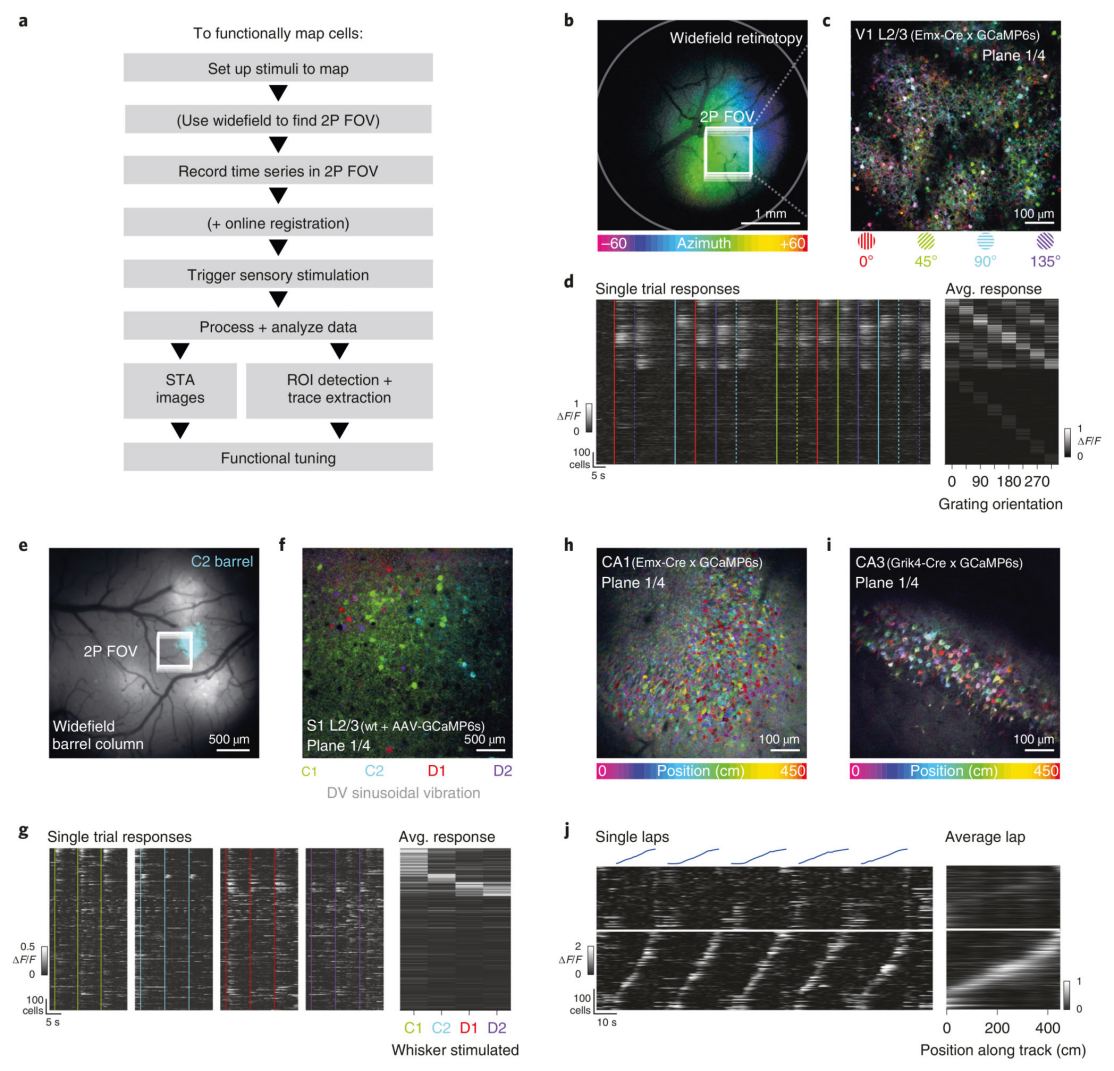
Mapping functional responses online. **a**, Example workflow for collection and fast online analysis of functional responses. Key optimizations that allow same-session analysis are (i) online motion correction in real time, which eliminates lengthy post-acquisition motion-correction times, and (ii) generation of stimulus-triggered average (STA) images, which intuitively map response strengths and tunings onto the spatial locations of cells. **b**, STA image generated from widefield calcium imaging data acquired in primary visual cortex (V1) as a contrast-reversing checkerboard (10°) drifted horizontally across a gray screen (25°/s) positioned in front of the contralateral eye. Pixels are colored by the azimuth that elicited the strongest response. The two-photon imaging volumetric FOV used for **c** and **d** is indicated. **c**, STA images generated from one plane in the two-photon imaging volumetric FOV indicated in **a** (L2/3 V1) as Gabor patches (30°) of drifting sinusoidal gratings (0.04 cycles/°) of four orientations (0°, 45°, 90° and 135°) were presented to the contralateral eye. Pixels are colored by the orientation that elicited the strongest response. **d**, Left, extracted traces showing single trial responses to stimuli indicated by vertical colored lines (color conventions are the same as in **c;** dashed lines are stimuli in the opposite direction to the solid lines). Right, average poststimulus response amplitude. Note that in both heatmaps, neurons have been sorted by preferred stimuli. **e**, Indicator expression image in primary somatosensory cortex (S1) (grayscale) overlaid with a thresholded STA image heatmap (cyan) acquired during vibration of the C2 whisker. The two-photon imaging volumetric FOV used for **f** and **g** is indicated. **f**, STA image generated from one plane in the two-photon imaging volumetric FOV indicated in **e** (L2/3 S1) as each of four whiskers were stimulated individually (C1, C2, D2 and D1). Note that this is a composite image combining data from four separate movies, one for each whisker. **g**, Left, extracted traces showing single trial responses to stimuli indicated by vertical colored lines (color conventions are the same as in **f**). Right, average poststimulus response amplitude. Note that in both heatmaps, neurons have been sorted by preferred stimuli. **h**, STA image generated from one plane in the two-photon imaging volumetric FOV in hippocampal CA1 (animal genotype: Emx-Cre × CaMKII-tTa × GCaMP6s). Data were acquired as animals ran along a virtual linear track. Color indicates the position along the virtual track that elicited the strongest response, and color intensity indicates the response magnitude. **i**, Same as **h** but in a hippocampal CA3 FOV (animal genotype: Grik4-Cre × CaMKII-tTa × GCamP6s). **j**, Left, extracted traces from neurons in **i** showing single trial responses (bottom heatmaps) as an animal ran laps along the virtual linear track (top trajectories). Right, response of all neurons averaged across laps. Note that in both heatmaps, neurons have been first divided into those that are spatially modulated (bottom) and those that are not (top) and sorted within those pools by preferred firing location (place field). Avg., average; ROI, region of interest.

**Fig. 8:**
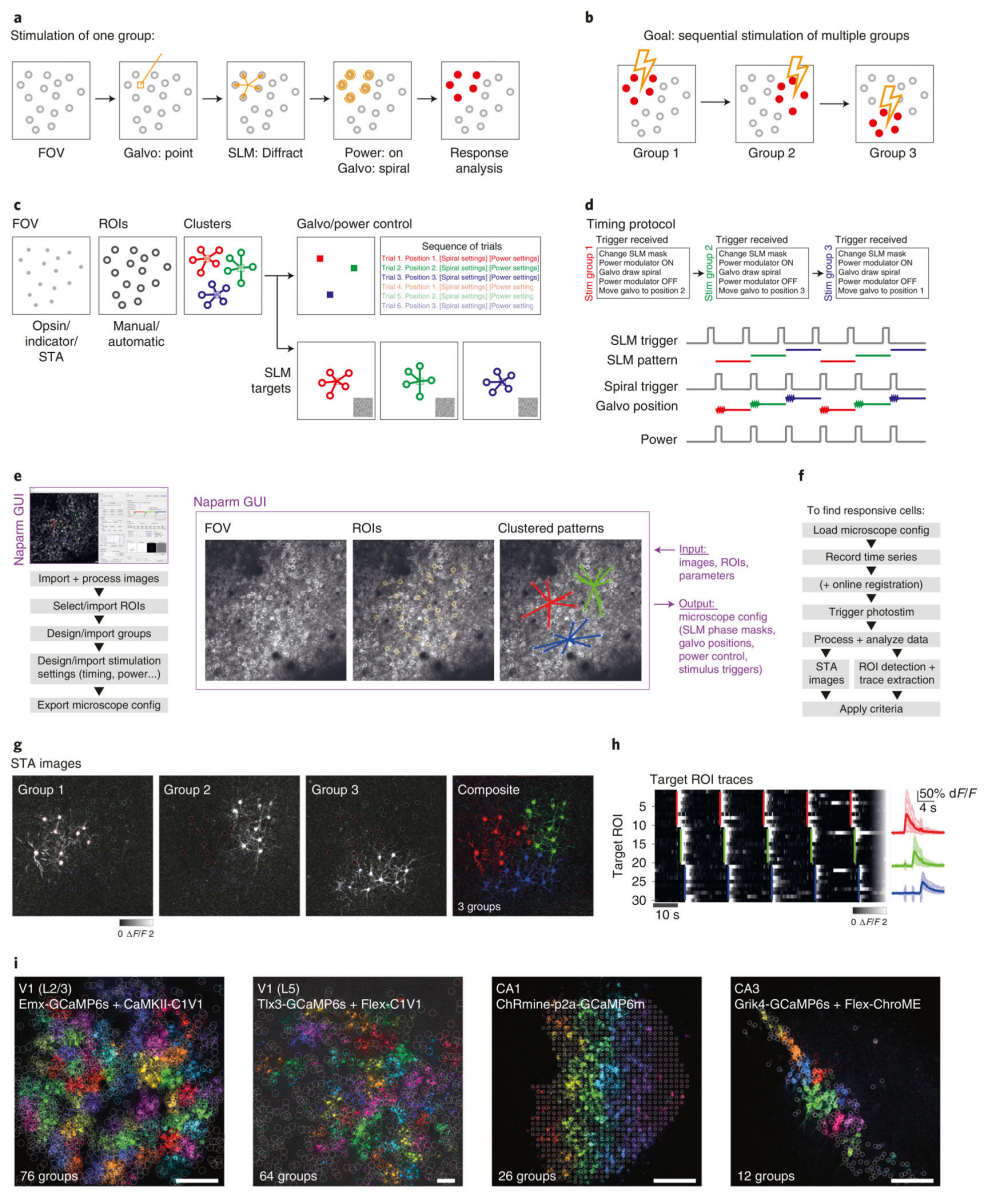
Mapping photoactivatable neurons. Panels **a** and **b** provide an overview, panels **c** and **d** show the workflow, panels **e–h** show a worked example and further examples are provided in panel **i. a**, To stimulate one group of cells, the galvanometer mirrors first move to the centroid of the target neurons. The SLM displays a phase mask resulting in diffraction of the beam focusing onto the cells of interest. To stimulate the cells, the power-modulation device permits light transmission and controls the intensity, and the galvanometer mirrors simultaneously move all the diffracted spots in a spiral over the cell bodies of interest. After stimulation, the response can be analyzed. **b**, To stimulate all cells in the FOV, sequential stimulation of smaller groups is required. **c**, The workflow. First, an FOV is loaded and analyzed, and ROIs are found/selected and then clustered into groups that will be stimulated one after the other. To drive the microscope system to perform the stimulation as described in a, various files are required to configure the subsystems, including the positioning of the galvanometer mirrors, the SLM phase masks and a trial sequence listing the stimulation order. **d**, A voltage command is sent from external hardware to trigger the delivery of a photostimulation. The trigger will update the SLM phase mask, move the galvanometer into position, turn on the power modulation and start the galvanometer spiral. **e**, Software used to design photostimulation mapping experiments. The workflow is as follows: loading FOV images, selecting ROIs, designing the grouping, configuring the stimulation parameters and finally exporting the files to load into microscope control systems. **f**, Protocol to run the photostimulation mapping experiment: load the generated microscope configuration files, record a time-series movie (optionally performing online motion correction) and trigger the stimulations throughout the recording. After acquisition, the data are analyzed to identify responsive cells. **g**, Example STA images of the response after photostimulation of three groups (stimulated 1 s after each other). The right panel shows a composite image in which the hue corresponds to pattern number and the intensity corresponds to the response magnitude. **h**, Activity traces extracted from ROIs targeted in the same experiment as in **g**; stimulations are indicated by vertical colored lines extending through the neurons that were targeted in a particular pattern. The right panel shows STA traces. **i**, Example STA images for the response to photostimulation mapping experiments as in **g** in various brain regions (labeled on each image). Colors represent groups of neurons photostimulated concurrently. config, configuration; Galvo, galvanometer.

**Fig. 9:**
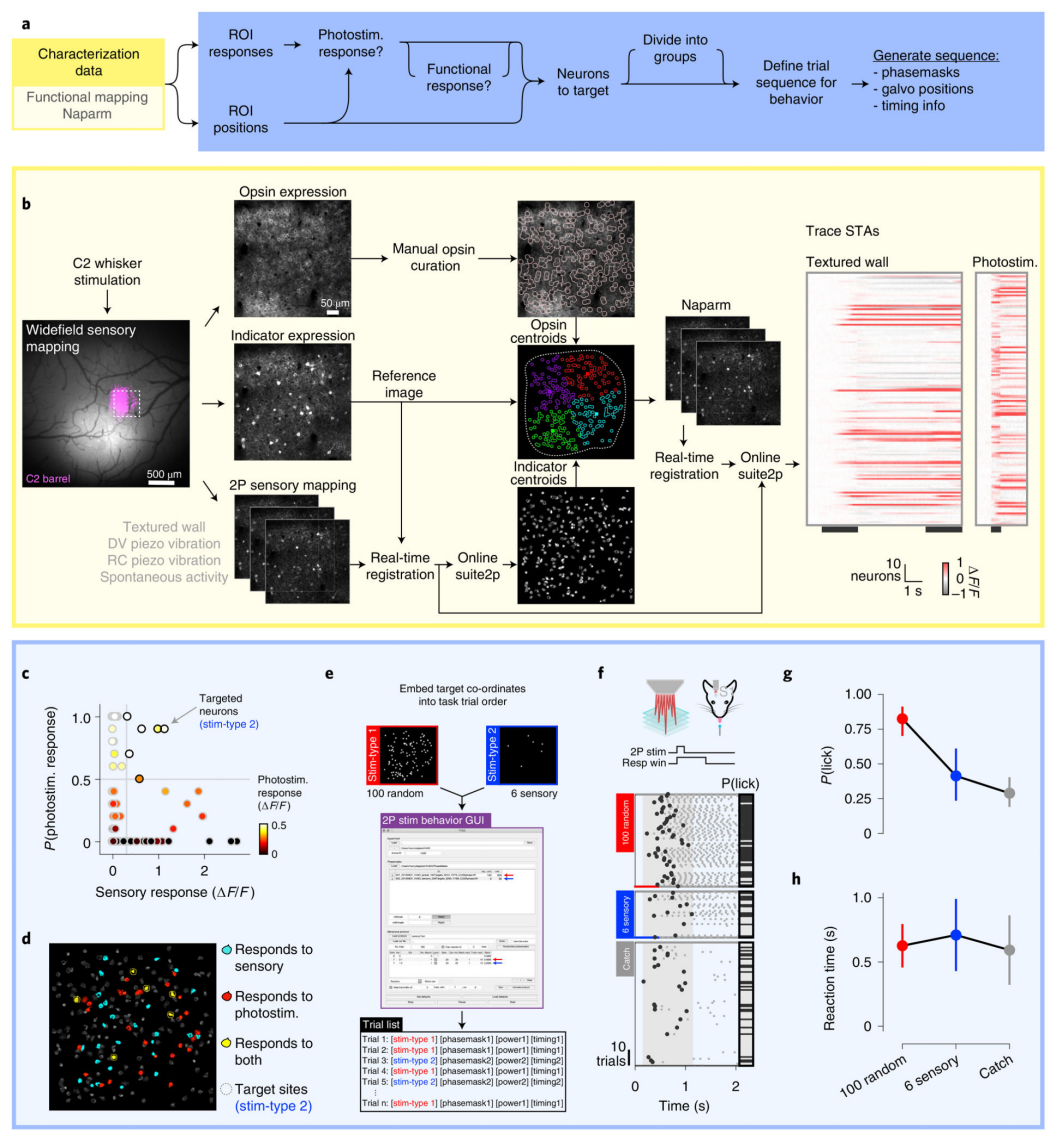
A worked example: probing the perceptual salience of sensory-responsive neurons in the L2/3 barrel cortex by using targeted two-photon optogenetic stimulation. **a**, Schematic illustrating the general workflow from acquisition of characterization data to generation of components necessary to perform an all-optical experiment. **b**, Sequence of steps necessary to acquire relevant characterization data in this worked example. Left, C2 whisker stimulation during widefield calcium imaging of S1 allows identification of the C2 whisker barrel used as the two-photon FOV going forward. Two-photon expression images of opsin (middle left, top), indicator (middle left, middle) and two-photon imaging movies acquired during whisker stimulation (middle left, bottom) are used for selection of opsin-expressing ROIs (middle right, top) as a reference image for real-time motion correction (middle right, middle) and to generate functional GCaMP-expressing ROIs via online Suite2p (middle right, bottom) with opsin and Suite2p centroids and then used to generate two-photon photostimulation targets (middle). Only targets in the central region of the FOV are included (dashed white border), and these are divided into four groups of 50 neurons (colors). These groups are then used for the Naparm protocol, which is subsequently concatenated with previously acquired sensory characterization movies and run through online Suite2p. Right, This yields extracted traces from Suite2p ROIs that can be used to generate sensory and photostimulus STAs. **c**, Thresholds (gray dashed lines) are set on the sensory and photostimulus responses to find photostimulable neurons that also respond to sensory stimuli. **d**, Overlay of sensory and photostimulus response types onto the Suite2p ROI image, highlighting the location of target neurons (dashed circles). **e**, Target coordinates for two photostimulus trial types, one stimulating 100 random neurons (trial type 1) and another stimulating just the 6 sensory-responsive neurons (trial type 2; see **c** and **d**), are embedded into a behavioral task paradigm via a custom GUI that allows the binding of specific phase masks with trial types and the organization of trial types into a sequence of trials for a given behavioral session. Note that panels are for schematic purposes; details will vary per experiment. **f**, Top, task schematic; mice are required to report the detection of two-photon photostimulation targeted to ensembles of neurons (500-ms duration; 10 × 20-ms spirals at 20 Hz) by licking at an electronic lickometer for sucrose rewards in a 1-s response window after the onset of photostimulation, or to withhold licking on catch trials during which no neurons were photostimulated. Bottom, sorted lick raster split by trial type from the behavioral session immediately after the characterization in **b–e**. Trials were delivered pseudorandomly but are sorted for display. Stimulus durations are shown as colored bars at the bottom of the raster. **g**, Proportion of trials on which the animal licked, and therefore putatively detected photostimulation, for each trial type. Error bars are binomial. *N* = 1 session; 58 random trials, 29 sensory trials, 78 catch trials. **h**, Reaction time for each trial type. Error bars are s.d.. *N* = 1 session; 58 random trials, 29 sensory trials, 78 catch trials.

In this protocol, we present a series of procedures to enable successful execution of all-optical experiments (Fig. 2), drawing on our experience with such experiments in five brain regions (L2/3 S1^14,36,40^, L2/3 V1^41^, L5 V1 (M.F., D.H., unpublished observations), CA1^43^ and CA3 (L.E.R., R.N., unpublished observations)). These procedures range from the setup and calibration of an all-optical system (Procedure 1; Figs. 3, 4), to the preparation of an indicator- and opsin-expressing (Procedures 2 and 3a; Fig. 5) and task-performing (Procedure 3b; Fig. 6) animal, to the characterization of functional (Procedure 4a; Fig. 7) and photostimulation (Procedure 4b; Fig. 8) responses in an FOV, and conclude with the design and implementation of an all-optical experiment (Procedure 5; Fig. 9). We present the various optimizations that are required for successful implementation of the strategy, as well as highlighting pitfalls and their potential solutions. We have also developed tools for optimizing the experimental workflow, including a strategy for mapping the photostimulation responses of all neurons in a given FOV (by using a software package that we call ‘Near-Automatic Photostimulation Response Mapper’ (Naparm)), as well as various other software tools used to control our behavioral and all-optical experiments. This protocol (including the expression strategies, calibration routines and software and hardware tools that we describe) has been instrumental in enabling all-optical experiments in our laboratory. In combination with the tools developed by other groups, the protocol can form the basis of a standardized toolkit to facilitate the dissemination of these techniques.

### Alternative methods

Previous implementations of simultaneous manipulation and readout of neural activity have been achieved by using either purely electrophysiological^46–50^ or low-resolution optical methods^51^, or a combination of both^52,53^. These methods have been unsuitable for ‘cracking the neural code’ on a large population level with sufficient flexibility and single-cell or single-spike resolution. Briefly, these methods are associated with a range of limitations, including manipulation artifacts on the recording channel, inadvertent activation caused by the recording or lack of spatial resolution. Solely electrophysiological approaches suffer from electrical artefacts in the recording channel during stimulation, the fact that recordings are invasive and the inability to target large groups of neurons while maintaining single-cell resolution. On the other hand, optical approaches have also suffered from a similar drawback because one-photon actuation stimulates many cells simultaneously (even when purposefully minimized^9^). In addition, using one-photon widefield excitation generates substantial autofluorescence throughout the tissue, which can generate a large optical artefact in the imaging channel, and moreover does not permit targetable manipulations with cellular resolution. Simultaneous two-photon holographic imaging and photostimulation methods relying on glutamate uncaging have been used only in vitro^54,55^. Combined optical and electrophysiology approaches also show promise for specific applications^56–58^, although it can be difficult to ascertain which neurons are being directly activated and which are downstream of the activated neurons^59,60^.

### Applications and future adaptations

Looking forward, the dissemination of the all-optical interrogation approach will crucially depend on the continuous development of more powerful hardware^16,37^, more intuitive software (this protocol and ref. ^41^), more accurate optical algorithms^61^ and more sensitive opsins^16,37^ and indicators^62^. Beyond these specific avenues for improvement, all-optical interrogation will also benefit from ongoing work to increase our ability to image deep in cortical tissue, through the use of three-photon imaging^63–67^, red-shifted indicators^68–70^, adaptive optics^71,72^ and gradient-index (GRIN) lenses^20,73^, as well as approaches that allow us to image more neurons^74–78^ at faster rates^79–82^. Finally, the continuous development of genetically encoded voltage indicators^83–86^ will hopefully pave the way to high-resolution all-optical electrophysiology of populations of neurons in vivo during behavior^84,87–89^.

### Limitations

We have successfully applied this protocol for all-optical experiments by using commercial Bruker^40,41^ and Thorlabs^43^ microscopes. Although the general principles will apply to any setup configured for all-optical experiments, our software routines (available with this protocol) may have hardware-specific implementations that could preclude their use with other systems. The all-optical approach, although powerful, has several limitations that should be carefully assessed in each experiment. Any experiment requiring the expression of exogenous constructs runs the risk of cytopathology due to excessive expression levels, and this risk is increased in all-optical experiments, given the need to express both a calcium buffer (the activity indicator) and membrane channel (the opsin) in the same neurons. Therefore, steps must be taken to monitor and mitigate overexpression while also ensuring sufficient expression for experimental purposes^14,23,24^. Users should also be aware of problems affecting animal health and welfare as well as aberrant patterns of neural activity that can arise from the use of transgenic animals^90^. The specifications of optical systems used for such experiments crucially dictate their experimental strengths and weaknesses. Therefore, we encourage users to rigorously characterize, maintain and report in publications the optical and physiological resolution of the system used for each experiment (e.g., as in refs. ^14–17,37^), as well as continuously maintain any necessary calibrations. This is important for ensuring consistency of results over time and reproducibility between laboratories, helping with interpretation of results and informing experimental design and subsequent analysis pipelines. It should also be noted that all-optical systems, including those described in this protocol, are subject to alignment drift over time, and therefore routines should be put in place to ensure that metrics indicating correct functioning are monitored frequently (although this need not always extend to the full rigorous characterizations described above). Photostimulation with two-photon excitation can cause heating and potential photodamage because of various linear and non-linear processes^16,91,92^, and therefore efforts must also be made to mitigate this by using safe levels of laser power throughout experiments and monitoring cell health during the experiment.

### Experimental design

#### System design

The ‘all-optical’ method combines genetic engineering of neurons, multiphoton imaging and holographic optogenetic manipulation for optical recording and targeted photostimulation. Integrated hardware and software are needed to coordinate all aspects of the system to read and write neural activity in awake, behaving animals. Building on previous work^12,93,94^, in this section, we provide an overview of the system design and then provide more detail in subsequent sections.

For light-based readout and control, neurons need to be engineered to co-express two proteins: a fluorescent indicator to record their activity and an effector (usually an opsin) to manipulate their activity. The absorption spectra of these two proteins should ideally be as distinct as possible, allowing for different wavelengths of light to selectivity excite each protein. We restrict our discussion here to two-photon excitation of both the indicator and the effector, because it provides good depth penetration, high spatial resolution and optical sectioning for both the read and write channels.

A core element of the approach is that two-photon imaging of large populations of neurons is performed simultaneously with two-photon stimulation of those or other neurons. This simultaneity is made possible by two independent light paths that can be combined and directed to the same FOV through a single objective. The imaging light path is that of a conventional two-photon scanning microscope. The second light path, for photostimulation, requires an additional laser source tailored to optogenetic stimulation, a power modulation device (a Pockels cell or an acousto-optic modulator) and a programmable diffractive element, typically a spatial light modulator (SLM), that allows the targeting of multiple neurons. After this SLM, there are two common configurations to couple the beam into the microscope, depending on the details of the targeting method. One common system (and one used by the authors), is to use a telescope to reimage the SLM onto a set of galvanometers, which themselves are conjugate to the back pupil plane of the objective. The galvanometer mirrors can then be used to ‘spiral’ the focused points of light over neuronal cell bodies to excite sufficient numbers of actuator (opsin) molecules to generate spikes in targeted neurons^28^. The other common system uses an additional grating or SLM and a different telescope arrangement to allow for temporal focusing^33,35,95^. These ‘serial’ and ‘parallel’ illumination strategies are compared in Box 1. In both cases, the optional second set of galvonometers in the photostimulation light path can also be used to effectively extend the addressable FOV of the SLM (which in itself is limited by SLM pixel count) by ‘steering’ the SLM center to different parts of the FOV about which beamlets can then be diffracted (Fig. 8). Finally, the photostimulation and imaging beam are combined into the common path of the microscope downstream of the respective galvanometers.

As mentioned above, SLMs are common to most all-optical setups. This is because they enable user-programmed diffraction of the stimulation beam into multiple beamlets targeted to many individual neurons at once. However, SLMs are not 100% efficient^96^, neither in their fill factor nor in their diffraction efficiency. As a result, some light remains undiffracted in the 0th diffraction order (‘zero order’) of the beam and should thus be blocked before the galvanometers to prevent nonspecific stimulation of neurons. To block this zero order, we position a small physical blocking element (because the incident power can be high and is localized in a focal point, we use reflective materials such as a piece of aluminium foil or lithographically printed gold deposits rather than absorbent materials) installed in a translatable mount at the focal plane of the SLM, preventing the zero order from propagating further through the rest of the system. Alternatively, one can mitigate the zero order optically by using a cylindrical lens^97^. Although we and several commercial systems use an SLM in combination with galvanometer spiral scanning to rapidly illuminate multiple cell bodies, this approach is not the only solution. Galvanometer mirrors, without an SLM, can be used to sequentially target single cells one at a time^13,19,20^. In addition, a diffraction grating, with or without an SLM, can be used to generate a temporally focused, arbitrarily shaped blob of light illuminating laterally extended areas (e.g., the whole soma) at once, which can be multiplexed in 2D and/or 3D via an additional SLM, negating the need to ‘spiral’ the beamspots^13,32,35,95^ (Box 1). As implied above, there is no single blueprint for designing an all-optical system. However, we provide a general system diagram for the workflow of our own system (Fig. 3), and both we and others have provided details of specific, working implementations in the past^13,14,16,17,19,21,35,37,38,45^ that can serve as resources for building one’s own system once the specific requirements are known.

In designing an all-optical system, it is critical to minimize ‘cross-talk’, the unintentional activation of opsin-expressing neurons by the imaging laser and/or the unintentional generation of fluorescence in the imaging channel by the photostimulation laser. This is achieved by choosing opsin and indicator combinations that require sufficiently different illumination wavelengths, excitation powers and dwell times. These factors pivotally constrain the design of an all-optical system’s imaging and stimulation light paths.

To perform an all-optical experiment involving targeting of functionally identified neurons, online analysis is required to identify, characterize and then stimulate the cells of interest (Fig. 3). For many experiments in systems with stable neuronal tuning to experimentally relevant variables^98–103^, it is possible to do analysis offline and revisit the same FOV on subsequent days to photostimulate neurons of interest identified in the intervening period^37^. This analysis is identical to that required for ‘recording-only’ experiments routinely performed by the field and will thus not be described here, excepting any details shared with the rapid online analysis routines discussed immediately below and in subsequent sections. However, for some experiments, it is necessary to rapidly perform the analysis immediately after the acquisition of data has completed while the animal remains mounted under the microscope. This is particularly important if navigation back to the exact same FOV over multiple days is difficult^40^, or in brain regions^104–109^ or for stimuli^110^ where the level of representational drift is such that neurons tuned to variables of interest may change from session to session. The speed of this analysis is vital for maintaining the performance of animals in behavioral experiments and is facilitated by real-time access to the incoming data stream; the raw imaging data are streamed to an accessible raw data file with minimal overhead for file operations (avoiding slow TIFF read and write overheads) while also allowing real-time, frame-by-frame motion correction as the data are being acquired. These optimizations allow for analysis of the neural data immediately after acquisition.

To identify recorded neurons and extract their activity through time, we currently use a version of Suite2p^78^ modified to work on the real-time registered binary files mentioned above or semi-automatically select regions of interest (ROIs) from pixelwise stimulus-triggered average (STA) images of the FOV responses to stimuli of interest. With access to the raw data stream, it is also possible to algorithmically identify ROIs as the data are being acquired^111^ and/or readout and manipulate activity by using a closed-loop approach^36,112^, allowing the real-time neural activity or animal behavior to guide the optogenetic manipulation. Finally, the choice of the pattern of targeted neurons to stimulate depends on three factors: physical location, functional identity (i.e., tuning to a task/stimulus variable of interest) and whether the neurons are responsive to photostimulation. Given these constraints, we construct a target pattern and generate requisite files to be loaded into the respective modules of the all-optical system that include the diffraction pattern to be made by the SLM, the instructions for the microscope software to position photostimulation galvanometer mirrors and control the photostimulation power modulator and triggers to synchronize the pieces of hardware. For subsequent analysis, we typically record signals of all imaging frame acquisitions, stimulation triggers and behavioral events by one master ‘synchronizer’ data acquisition device.

#### Choice of indicator/opsin combination

All-optical constructs should be carefully chosen and evaluated to balance the sensitivity of the indicator for reporting spikes and the efficacy of the opsin for two-photon activation, while minimizing cross-talk, usually by ensuring maximal spectral distance between the two excitation wavelengths. Cross-talk comes in two main forms. First, there is the unintentional activation of opsin-expressing cells by the laser used to image the activity indicator. Second, there is the unintentional excitation of the indicator by the photostimulation laser. The level of such cross-talk is determined by the overlap (peak location and width) of each construct’s excitation spectra. Most existing all-optical setups have relied on widely used green indicators (which are excited by blue one-photon light), such as those of the GCaMP family^24^ (optimal two-photon λ_excitation_ ~920 nm), combined with far red–shifted opsins such as C1V1^113^ or Chrimson^114^ (two-photon λ_excitation_ ~1,000–1,100 nm). This is because green indicators continue to exhibit better signal-to-noise ratio (SNR) than red indicators. These setups achieve minimal cross-talk in most use cases^13,14^ through sufficient spectral separation of peaks in the excitation spectra of the indicator/opsin and relatively low opsin sensitivity. Slightly less red-shifted opsins, such as ChroME^16^ and ChRmine^37^ (two-photon λ_excitation_ ~870–1,200 nm), have also been used with green indicators because of their improved speed and sensitivity, with the caveat that imaging power and dwell time must be kept sufficiently low^16^ because of the increased overlap and sensitivity. Although these green indicator/red opsin combinations are common, it should be noted that the spectra of many red-shifted opsins have an appreciable shoulder toward shorter wavelengths^29,113,115–117^ that potentially allows them to be activated by the imaging laser. To further optimize this, several groups have begun to combine red indicators (which are excited by yellow one-photon light), such as jRCaMP or jRGECO^70^ or xCaMP^118^, with blue-light sensitive opsins^17,119^ such as ChR2 or ChroME. In addition to distance between peaks, these combinations benefit from less overlap across the entire spectrum, something that may become particularly important when using all-optical approaches in deep regions that require higher laser power (something that will also benefit from red-shifted indicators). Such red indicator/blue opsin combinations will benefit from future improvements in red-shifted indicator SNR and increased availability of low-repetition-rate lasers of appropriate wavelengths for blue opsins.

Different experiments may require opsins with different features. For experiments in which spike timing with millisecond precision or specific spiking rate are important variables, opsins with fast rise times, and critically, fast off channel kinetics (on the order of 5–10 ms) are required to ensure high temporal fidelity (such as Chronos or ChroME). Alternatively, if the total number or simply the identity of cells is important, with less emphasis on temporal features of their activity, opsins with slower temporal characteristics (e.g., C1V1) can be used. It is important to note that there is typically a trade-off between opsin channel open time and sensitivity. Because the channel open time of fast opsins is short, and potentially too short for a sufficient number of channels to remain open for the duration of a spiral scan, area illumination may be required because it leads to the simultaneous opening of many channels (with associated lower latency and jitter), which is more likely to lead to sufficient depolarization for the targeted neuron to spike. That said, modern opsins can be made sufficiently sensitive that near-millisecond precision can be achieved with spiral scanning^37^. In addition, in this protocol, we provide examples in which we have extended the genetic engineering approach to use the Cre/LOX system, relying on recombinase-expressing transgenic mice to restrict expression to specific cell types of interest. Finally, although most all-optical protocols have focused on activation by using cation channels, it should be noted that it is also possible to perform targeted all-optical suppression with two-photon recruitment of anion pumps such as eArch3.0^29^ and, more recently, potent anion channels such as GtACRs (*Guillardia theta* anion-conducting channelrhodopsins)^16,17^.

As with excitatory opsins, relevant opsin characteristics such as photocurrent magnitude and off-kinetics need to be tailored to the specific experimental goal.

Opsin molecules are typically co-expressed with a static fluorescent marker to visualize expression. These markers can either be directly fused to the opsin in the membrane (e.g., C1V1-Kv2.1-mRuby) or be separate from the opsin molecule through the use of self-cleaving peptide links (e.g., C1V1-p2a-mCherry) resulting in the opsin remaining in the membrane, but the fluorophore free in the cytosol. The reporter fluorophore can also be restricted to the nucleus, which can help in cell identification (e.g., ChroMe-p2a-nls-mRuby). Finally, the opsin can be expressed from a bicistronic construct that also contains the indicator gene and thus requires no additional fluorophore for identification (e.g., GCaMP6m-p2a-ChrMine).

One major determinant of the fidelity, or spatial resolution, of photostimulation is the localization of opsin expression. If opsin is expressed throughout the neuron, in the membranes of all its processes, photostimulation directed far away from the soma may still depolarize that neuron if one of its processes happens to be in the stimulation volume. To this end, researchers have developed somatic restriction strategies^16,19,37,39,119–121^, whereby opsin molecules are localized to and concentrated in the soma and proximal dendrites, rather than throughout the dendritic arbor and axons.

The optogenetic toolbox is continually evolving, which precludes including a complete or definitive list here. Fortunately, concerted efforts by many groups are constantly yielding up-to-date, useful resources detailing opsin spectral responsivity (relevant to laser choice and cross-talk), sensitivity (relevant for excitation paradigm) and kinetics (relevant for timing, photostimulation strategy and cross-talk)^29,122–126^ that can be used for making informed decisions about which construct is appropriate for a setup.

After having chosen an opsin and indicator pair, the next critical step is to optimize the level of expression. Achieving balanced co-expression is challenging, perhaps due to promoter-specific differences in different brain areas, but also probably due to overexpression of one or both constructs^23,24^. Because of factors like competition between different viruses, and depending on the experimental goal of long-term health versus maximum possible expression, all combinations of chosen indicator and opsin must be thoroughly tested in the particular preparation of interest at a range of titres over different timescales to assess efficacy (avoiding underexpression) and long-term health (preventing overexpression^23,24^; see also Supplementary Fig. 8 in ref. ^14^). Some promising combinations that simplify this issue are to combine virally expressed opsins with transgenic GCaMP indicator mice^16,40,41^, to use a single bicistronic virus encoding both indicator and opsin simultaneously^37^ or to generate transgenic mice that co-express both opsin and indicator^127^. As a guide, we also provide a table detailing key all-optical combinations that have been published (Supplementary Table 1).

#### Choice of photostimulation laser

**Fig. 10:**
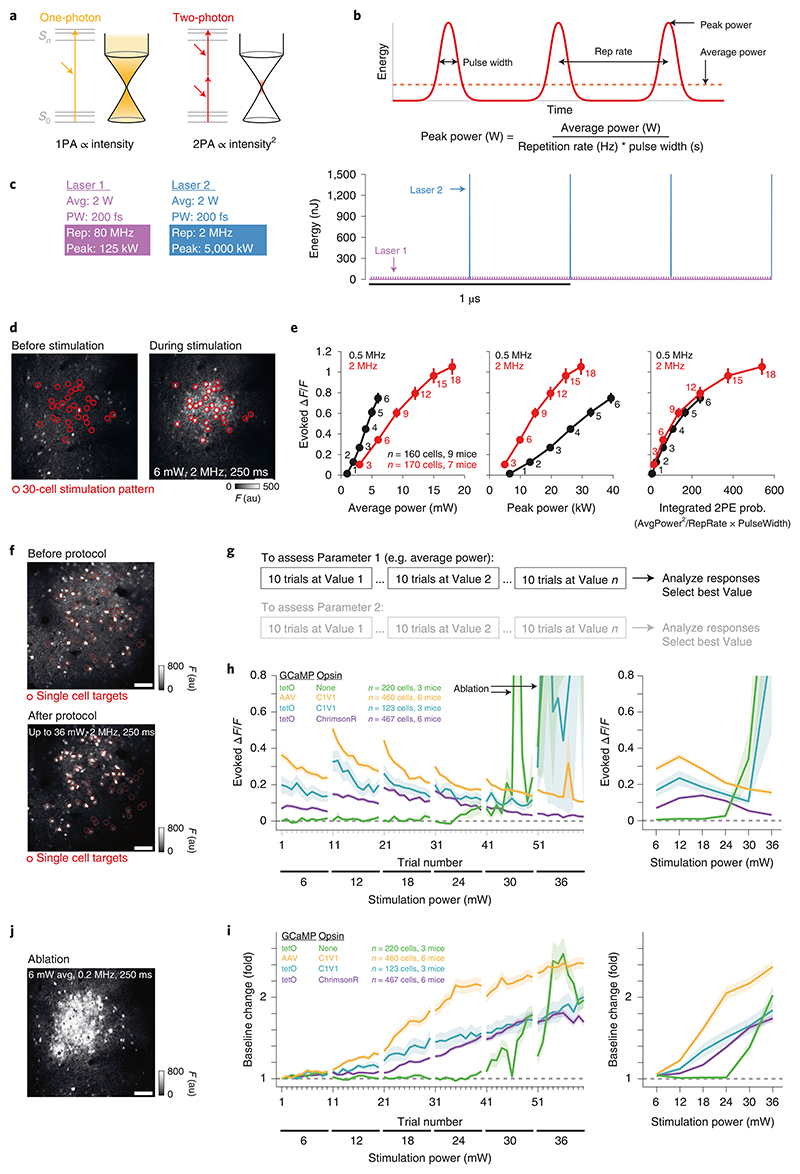
Two-photon excitation and calibration of safe and effective stimulation parameters. Panels **a–c** show laser terminology, panels **d** and **e** show photostimulation efficiency and panels **f–j** show photostimulation safety. **a**, Two-photon excitation compared to one-photon excitation. b, Diagram illustrating key parameters of pulsed lasers used for both imaging and photostimulation applications. **c**, Hypothetical comparison between two lasers with the same average power and pulse width, but with a different repetition rate (and thus peak power). **d**, FOV fluorescence image before and after photostimulation of a 30-cell ensemble. Animals were wild types virally expressing AAV1-hSyn-GCaMP6s and AAVdj-CaMKII-C1V1. Scale bar represents 100 μm. **e**, Stimulation of 10-cell groups (prefilter for responsive neurons) with range of average powers at two different laser repetition rates (indicated in red and black). These groups were stimulated in blocks of increasing powers at one of the two repetition rates. Spiral parameters were as follows: 15-μm diameter and 20-ms duration repeated 5 times at 20 Hz. 10 trials of each stimulation were conducted, with 10 s between each stimulation. Plots show the average response size (Δ*F*/*F*) of the targeted ROIs as a function of the average power per cell (measured on the sample), the peak power or the integrated probability of two-photon excitation. Numbers indicate the average power (mW). On the basis of these assessments, 6 mW (3 mW/µm^2^) per cell (at 2 MHz) was chosen as an effective power to use for our stimulation experiments, given its ability to evoke reliable transients while being safe to not cause noticeable damage (see **f–i** below). **f**, To assess phototoxicity, we stimulated single cells with increasing average powers (from 6 to 36 mW at 2-MHz repetition rate). Cells were selected from GCaMP expression images with no knowledge of opsin expression. Various expression strategies were used. tetO refers to transgenic animals expressing GCaMP6s under the tetO system^134^. Spiral parameters were as follows: 15-μm diameter and 20-ms duration repeated 5 times at 20 Hz. 10 trials of each stimulation were conducted, with 10 s between each stimulation. Images show the FOV of GCaMP expression before and after the single-cell stimulation protocol. Note the bright filled appearance of the targeted cells after the protocol, indicating that damage occurred by the higher stimulation powers. Scale bar represents 100 μm. **g**, Outline of the calibration protocol. We select one parameter at a time, keeping all others constant. We perform 10 trials at a given value of that parameter and then increment it and acquire 10 more trials, repeating until we reach the maximum value to be tested. Subsequent analysis is used to select the power that was effective (resulted in activation of neurons) and also safe (no changes in baseline fluorescence). If there are multiple parameters to be tested, we would then proceed in a similar fashion with the next parameter. **h**, Average response size of the targeted cells across trials and blocks of increasing power. Responses all tend to decrease within a block, probably because of opsin desensitization. The large ‘responses’ at 30 and 36 mW (22 and 26 mW/µm^2^) are a result of photoablation. **i**, Average baseline fluorescence of the targeted cells across trials and blocks of increasing stimulation power. We used the baseline fluorescence as an indicator of cell health, with increases (that were not the tail end of GCaMP transients) representing an undesirable change. Note the strong increase after a few trials at 18 mW (10 mW/µm^2^), indicating that damage is accumulating in the targeted cells with this stimulation power. In this panel, it should also be noted that we find that opsin expression lowers the power threshold required to cause increases in baseline brightness (c.f. green *TetO* + *None* data with all other conditions). To our knowledge this has not been reported before, but it is possible that this results from the impact on cell health caused by the increased metabolic load imposed by additional opsin expression. **j**, An example of intentional photoablation of a multiple-cell ensemble, by using a very-low-repetition-rate (0.2 MHz) laser. 2PE prob., two-photon excitation probability.

It is important to choose the photostimulation laser carefully to optimize the activation of opsin-expressing neurons. The two-photon absorption by opsins (and thus activation of neurons) is proportional to the square of the excitation light intensity (Box 2; Fig. 10), and the optimal stimulation will be achieved with wavelengths closer to the peak of their excitation spectrum. Pulsed lasers for two-photon photostimulation are described by a few key parameters: wavelength, peak power per pulse, pulse repetition rate and pulse width (which together dictate the average power). We discuss these parameters in detail below.

Laser wavelength for photostimulation (either when selecting a fixed-wavelength laser or when using a tunable laser) ideally should be chosen with respect to the two-photon spectra of the indicator and opsin to maximize the spectral separation, and thus minimize cross-talk, between them. In practice, however, given the prevalence of low-repetition-rate lasers in the >1,000-nm range, opsins are often chosen to match available lasers. The laser power at the wavelength closest to the peak of the opsin’s two-photon excitation spectrum will dictate the number of neurons that can be activated simultaneously; the total available power on sample (the light that is focused under the objective, consisting of the diffraction pattern not blocked by the zero-order block) will be divided (usually equally) among the holographically split beamlets targeted to individual cells.

Pulsed lasers used for two-photon imaging typically operate at a high repetition rate (~80 MHz), which is necessary given the speed at which the focused beam is scanned across the tissue of interest, dictating the ‘dwell time’ for fluorophore exposure. This high-repetition pulse rate is associated with a trade-off between the peak power—power delivered by each pulse—and the time-averaged power (Box 2). Although similar lasers—Fianium (2 W of average power, 80-MHz repetition rate) and Coherent Fidelity (2 W of average power, 80-MHz repetition rate)—have been used for photo-stimulation of opsin-expressing neurons^13,14,30,36,42^, we and other groups have found more success with a different type of laser. Low-repetition-rate lasers—e.g., Amplitude Satsuma (1,060 nm, 20 W of average power, 0.5/2-MHz repetition rate); see also Coherent Monaco, Menlo BlueCut, SpectraPhysics Spirit and alternatives—are associated with a much larger peak power (compared with high-repetition-rate lasers) while maintaining a similar average power at the sample^16,36,37,39–41,119^. High peak powers more efficiently activate opsin molecules by means of two-photon absorption, while opsin and cellular integration kinetics allow for the reduced frequency of pulses. Using pulses with higher peak powers means that less average power is required to successfully stimulate a cell. Less average power translates to less thermal energy and thus less heating of the tissue (which could lead to thermal damage such as protein denaturation)^91,92^. At a given average power, higher peak powers enable the beam to be split across more neurons to be stimulated simultaneously. Note, however, that high peak powers can be associated with nonlinear damage mechanisms^128,129^. Our average power per cell (excitatory cortical L2/3 cells expressing C1V1 opsin) of ~6 mW (3 mW/µm^2^) at a repetition rate of 2 MHz was selected as a compromise between good photostimulation efficiency and minimal photodamage in our experimental configuration (Box 2). For stimulation times <40 ms (such as we use), this excitation power should give at most a temperature rise of 0.3–0.5 K for single neuron excitation^92^. This compromise depends on several parameters, including the sensitivity of the opsin and the imaging depth in tissue, and should be tested for each setup (e.g., for our deeper cortical L5 experiments, we typically use ~12 mW (7 mW/µm^2^) per cell at 1-MHz repetition rate).

#### Choice of two-photon photostimulation method

The high resolution of photostimulation with a two-photon beam spot comes at the price of a very small activation volume (a region of membrane on the order of the point spread function (PSF)). To increase this volume sufficiently to cause large enough depolarization and therefore generate action potentials, one can either use spiral scanning or beam-shaping with temporal focusing (Box 1). We have used spiral scanning^28^, which involves scanning the beam spot over the somatic membrane, because this strategy requires less average power to cause neurons to spike^45^. Beam-shaping (by underfilling an objective or using computer-generated holography or generalized phase contrast) allows users to increase the lateral extent of the two-photon PSF to be the size of a neuron, but at the cost of also increasing the axial extent (beyond the size of a neuron), which will degrade the resolution of stimulation. Temporal focusing (via a diffraction grating placed in the beampath) can improve the axial extent of the PSF by ensuring that the laser pulses have the shortest duration at the focal plane of the objective, with the pulses broadening rapidly along the axial direction. The short pulses at the focus provide greater two-photon absorption relative to those out of focus, confining the excitation volume and recovering stimulation resolution.

#### Patterned illumination device

We use a reflective phase-only liquid crystal on silicon (LCoS) SLM to introduce phase changes across the wavefront of the photostimulation laser (described in detail below). The subsequent diffracted light is focused into spots of light on the sample targeted to specific neurons. We then use a pair of galvanometer mirrors to scan these spots of light over the cell bodies of these neurons in a spiral pattern to illuminate as many cell membrane–localized opsin molecules as possible.

The LCoS technology underpinning the functionality of reflective phase-only SLMs allows them to modify the phase of the wavefront reflected off them^130,131^. They achieve this via an active surface consisting of a layer of birefringent liquid crystals (functionality described below) between a ‘pixel array’ of transparent electrodes and a backplane. The voltage on each pixel controls the orientation (and thus refractive index) of the liquid crystals between them. Thus, depending on the orientation of the crystals (controlled by the voltage applied to each electrode), the resulting differences in the refractive index across the SLM surface result in variations in the effective optical path length of the light passing through those crystals, yielding modified phases in regions of the wavefront relative to the incident beam. This phase-modulated wavefront is then Fourier-transformed by the objective into multiple foci in the sample. The overall effect of the SLM on the laser wavefront is governed by the coordinated action of all its independently addressable pixels, the voltages of which are controlled by addressing the SLM with a ‘phase mask’ (the pattern of voltages applied across the SLM’s active surface, also referred to as a hologram, dictating the pattern of phases in the reflected wavefront). These phase masks can mimic the action of physical optical elements such as lenses to focus light or diffraction gratings to diffract light at a particular angle. In practice, these customizable phase masks are typically generated with a variant of the Gerchberg-Saxton algorithm^132^, an iterative Fourier, inverse-Fourier transform procedure (computation of which can be performed on GPUs), but other methods are available^61^.

There are several operating characteristics to consider when selecting an SLM:

1. *Overall efficiency*. Most SLMs do not have 100% fill factor; nor do they have 100% diffraction efficiency, and thus some power is lost by using them. Modern devices are specified as ~70–90% efficient, although this efficiency is reduced at wider angles of diffraction (the supplement of ref. ^133^ discusses this in detail). The light that is not diffracted remains in what is called the ‘zero order’ (which is blocked before entry to the objective).
2. *The size of the individual pixels*. Smaller pixels (keeping the SLM size fixed) will allow for greater diffraction angles to be achieved—increasing the size of the addressable FOV under the objective—but may come at the cost of cross-talk between neighboring pixels, resulting in reduced diffraction efficiency and thus a less efficient hologram.
3. *The size of the SLM*. This dictates the amount of de/magnification necessary to propagate through the rest of the optical system. The level of magnification will affect the size of the beam at the objective back aperture, thus affecting the effective numerical aperture (NA), while at the same time higher magnification will result in smaller diffraction angles being achieved by the objective^133^. There is therefore a trade-off between addressable FOV size and optical resolution.
4. *The speed of the SLM*. The speed at which the pixels can be driven to a new voltage setting (refresh rate of the driving electronics) as well as adopt a new voltage setting (liquid crystal settling time, i.e., the time for the liquid crystals to reorient to the newly applied voltage) will together dictate the rate at which new diffraction patterns can be focused on the sample^134–138^, which in combination with exposure durations (see ‘Choice of indicator/opsin combination’) will dictate the speed at which sequences of activity can be ‘played in’. Recent work simultaneously engineering opsins with faster kinetics and SLMs with more rapid refresh rates is yielding promising advances that are directly applicable in all-optical interrogation^37^.

Alternative systems for patterned illumination can be used. Galvanometer mirrors alone are appropriate if one needs to stimulate only one cell at a time^13,19,20^, whereby the focused beam spot can be steered to individual locations in sequence. Digital micromirror devices can also be used with one-photon illumination to target multiple locations simultaneously^139^, as an alternative to SLMs. Digital micromirror devices can be driven much faster than current LCoS SLMs (e.g., >1 kHz); however, they cannot be used with two-photon excitation because they have a lower damage threshold and lower power efficiency than SLMs (light that would fall on untargeted regions is discarded rather than refocused to the desired target locations), are unable to pattern light in 3D and have relatively poor axial resolution.

#### Choice of two-photon stimulation parameters

To provide precise control of the photostimulation of neurons, we can adjust the average power per cell, the duration of a single exposure and the timing of trains of exposures (inter-exposure interval as well as the total duration). For spiral scanning specifically, our photostimulation method of choice^14^, some key spiral parameters to consider are (i) spiral duration, increases of which will increase the number of evoked action potentials per spiral, as well as influence response latency and jitter; (ii) repetition frequency and number, increases of which will potentially result in reduced spiking reliability if they exceed parameters allowed by a given opsin’s kinetics and increased heating due to longer illumination times; and (iii) number of turns within a spiral (revolutions), increases of which will result in more opsin molecules excited per spiral, and thus potentially more reliable activation, but may also result in more desensitization due to increased excitation volume saturation and heating due to a more spatially confined excitation volume. For photostimulation response mapping using the relatively slow opsin C1V1 (τ_off_ = 40 ms), our most commonly used opsin, we generally photostimulate with a 20-ms spiral repeated 10 times at 20 Hz, resulting in a 500-ms stimulus epoch. These spirals are approximately a cell diameter in size (on the order of 10–15 µm), consist of three revolutions and an average power on sample of 6 mW (3 mW/µm^2^) per cell (with a 2-MHz repetition-rate laser). The stimulation parameters (pulse repetition rate and average power, keeping the stimulus duration constant) are chosen after careful calibration, ensuring efficient activation but also minimizing any signs of photodamage (in particular, increases in baseline fluorescence of stimulated neurons, which can be a sign of nonlinear ablation damage; see Box 2). In our calibration protocol, we varied only one parameter at a time and started at a lower bound for the value of that parameter, by using either optical or electrophysiological readout of neuronal activation (with electrophysiology being more sensitive but more challenging and generally limited to a single neuron in a given FOV). We typically stimulate in a given condition for 10 repeats, then increment the parameter and stimulate 10 more times, repeating until we have reached the upper bound of the given parameter. Then, by analyzing the resulting data, we can arrive at the optimal value for that parameter that gives adequate activation and minimal signs of damage. Our chosen values correspond to a stimulation rate considerably above the median firing rate of neurons in (vS1) cortex while also being within the physiological range of pyramidal cells over short time periods. The stimulation parameters should ideally be assessed for every system and opsin used to ensure effective but safe stimulation. The duration used for a single spiral scan depends on the sensitivity of the opsin used (i.e., the two-photon cross-section and the size of the induced photocurrent), the intensity of light used during exposure and the off-kinetics of the opsin. New, faster and more sensitive opsins such as ChRmine^37^ are compatible with much shorter spirals (approaching submillisecond exposures).

Another important characteristic of opsins is their off-kinetics. Slower opsins (such as C1V1 compared to ChroME) take comparatively longer to close after opening, meaning that they are unable to elicit another action potential as faithfully. These slower opsins will not be able to follow high-frequency (>40 Hz) trains of stimulation with as much fidelity, as the long *τ*_off_ blurs precise temporally patterned input, although their ability to integrate current over time can mean that they require lower powers to generate sufficient depolarization to drive neuronal spiking. In addition, all opsins have the potential to desensitize (i.e., become unable to open again after exposure). However, this can be partially mitigated if the stimulation volume is not saturating (i.e., activating all available opsin molecules), because the supply of as-yet-unexcited opsin molecules in the excitation volume can be subsequently recruited at later times throughout the stimulation epoch, effectively replacing the opsin molecules that have already desensitized to continue carrying current and depolarizing the cell. The off-kinetics and the rate of desensitization necessitate thought into both how frequently neurons are stimulated within a trial and the time between trials.

Because all these stimulation parameters are under the experimenter’s control, the stimulation can be designed to be physiological (replicating naturally occurring firing rates or ensembles of cells) or not, depending on the experimental question. We find it sufficient to calibrate our photostimulation parameters once per opsin/indicator combination on each system (assuming nothing significant changes over time) and use these for all subsequent experiments, although this relies on rigorous expression checking and subsequent exclusion of animals that are under-/overexpressing either construct from further experiments (see Procedure 3a). However, it should be noted that other groups have used online optical calibration to tailor some photostimulation parameters (such as laser power) to each group of targeted neurons^16^.

#### Characterization of the all-optical system

There are three main issues to consider when evaluating the performance of an all-optical system for use in biological experiments. These are (i) the achievable resolution of photostimulation (i.e., the specificity with which single neurons can be targeted without affecting their neighbors), (ii) the amount of cross-talk between the imaging and photostimulation channels (i.e., the degree to which the laser used to record activity in the FOV also excites the opsin (causing prolonged elevations in baseline spiking rate)^13,14,16,45^) and (iii) the degree to which the photostimulation laser generates fluorescence in the imaging channel (the photostimulation artefact, an increase in background brightness with swift onset/offset coincident with photostimulation)^16,37,40,45^. All these considerations depend on both optical and biological factors.

The resolution of photostimulation is determined by the effective NA of the photostimulation light path, which is set by the size of the beam at the back aperture of the objective. The degree to which the photostimulation beam fills the back aperture of the objective represents a trade-off with the maximum achievable diffraction angles, with more magnification resulting in shallower angles. The optical resolution of the system should be assessed empirically by using a sample of small (<1 µm) fluorescent beads to reconstruct the PSF by following standard procedures. As discussed above, a large determinant of the effective functional resolution is also biological in nature: the expression pattern of the opsin. Neurons are axially extended structures, and therefore if opsin is expressed through the extent of a neuron, this can exacerbate poor axial resolution. Enriching the opsin in the somatic and perisomatic compartment (somatic restriction, as discussed above) can mitigate this concern. To accurately quantify the resolution of the system, we recommend performing patch-clamp recordings of single neurons to provide electrophysiological ‘ground truth’ for photostimulation^14,16^. This involves delivering photostimuli at various lateral and axial offsets with respect to the cell body of the electrophysiologically recorded neuron. The resulting dataset can be used to construct a curve of action potential activation as a function of offset from the soma, and from this curve we can calculate the full width at half maximum (FWHM), a standard measure of the resolution (or PSF) of optical systems. It is important to note at this point that the FWHM for driving spiking depends on the amount of power and the fraction of opsin molecules needed to drive a spike: with more power, the FWHM is broader as you approach saturation of the opsin molecules in the excitation volume. Therefore, if one needs to saturate all opsins to drive spikes, the FWHM will end up being broad. As a result, strong, high-conductance opsins are generally preferable for confining the photostimulation FWHM to within acceptable ranges for targeted photostimulation. Using this measurement, we can subsequently define regions around the targeted sites in all experiments to either include or exclude neurons from data analysis on the basis of whether they might have been directly stimulated. This is particularly important for studies examining the synaptic recruitment of other, non-targeted neurons in the local neural circuit. Note that similar resolution measurements can more easily be obtained optically rather than electrophysiologically (as has been done in some studies^13,19,20,40^), but the readout of single action potentials is less accurate when using optical methods when compared to electrophysiological methods, and it provides no readout of subthreshold depolarization^14,16,21,29,37^. In general, we recommend initially calibrating a new system electrophysiologically and then performing regular optical calibrations to ensure that its functionality is maintained. Optical readout can be used on a trial-by-trial basis to ensure that photostimuli in a given experiment are working as intended.

The cross-talk between the imaging and stimulation pathways is largely set by the excitation spectra and sensitivity of the opsin molecule. If the opsin used is highly sensitive and/or has a spectrum that overlaps with the imaging wavelength, the imaging laser may activate the opsin and thus change the resting potential. Care needs to be taken to minimize the imaging laser exposure time or exposure intensity, for example, by using the minimum imaging laser power that results in usable data. In addition, by scanning volumetrically (in 3D), we can reduce the time the imaging laser is focused on any given cell, thereby reducing the time the opsin molecules are potentially excited. To accurately quantify the degree of cross-talk in a new system, we again recommend performing electrophysiological recordings of an opsin-expressing cell. By imaging the FOV at increasing imaging laser powers while recording the opsin-expressing cell, it is possible to assess how much the resting potential or firing rate changes as a function of the imaging laser. By testing various configurations (scanning speed, FOV size, volumetric scanning and illumination power), it is possible to design an imaging condition that has minimal impact on the baseline properties of opsin-expressing neurons.

The cross-talk between the stimulation and imaging pathways, which leads to artefactual increases in brightness in the imaging channel during photostimulation epochs, depends on the overlap between photostimulation laser wavelength and indicator/endogenous fluorophore excitation spectra, baseline brightness of indicator expression and high photostimulation powers (typically associated with simultaneous stimulation of many neurons). These increases in brightness have rapid onset/offsets that are coincident with photostimulation onset/offsets and typically corrupt pixels across the entire imaging FOV, often in a mesh grid pattern dictated by differences in pulse rate between the imaging and photostimulation lasers^16,133^. It is crucial to characterize the spatial extent and temporal characteristics of the photostimulation artefact on any new system and map how it relates to photostimulation power, number of photostimulation targets and typical levels of indicator fluorescence. Because the photostimulation artefact is present only during photostimulation, photostimulation epochs are brief and infrequent and the decay of most calcium indicators is long, a simple way around this issue is to exclude photostimulation periods from analysis and instead quantify neuronal responses in the period immediately after photostimulation offset^40,41,45^. However, if this is not possible (e.g., if photostimulation epochs are long) or undesirable, then several groups have developed hardware^16^, software^37^ and analysis^43,45^ methods to subtract the photostimulation artefact and enable analysis of data collected during photostimulation.

#### Choice of brain region

The all-optical protocol reported here can in principle be performed in any structure that is accessible to two-photon microscopy. We have carried out successful experiments in L2/3 vS1, L2/3 V1 and L5 V1, as well as hippocampal regions CA1 and CA3 (through an implanted cannula). All-optical interrogation of neural circuits has also been applied in the olfactory bulb^21^, orbitofrontal cortex (through a GRIN lens)^20^ and anterior lateral motor cortex^42^. The main region-specific considerations are that deeper areas will require higher laser power for both imaging and photostimulation and that the genetic identity of cells in different regions might preclude the use of some promoters for indicator/opsin expression. Areas deeper than ~500 µm are typically accessed through an implanted cannula^140^, by using GRIN lenses^20,73^ or with three-photon imaging^63–65,67^.

### Expertise needed to implement the protocol

The setup, maintenance and use of all-optical microscopes requires considerable optical expertise. Primary scope users should have the responsibility of maintaining and calibrating the microscope on a daily basis. They should be familiar with the full light-path alignment procedure, and they should be experienced in diagnosing and correcting misalignments. Beyond this, any user of the protocol described below requires minimal expertise beyond the animal handling required for the particular experiment being carried out and an ability to operate basic imaging and photostimulation functionality in the microscope software.

## Materials

### Biological materials

- Wild-type mice (C57/BL6; Charles River)
- Transgenic (expressing indicator, opsin or a recombinase) mice: Emx1-Cre (https://www.jax.org/strain/005628), CaMKIIa-tTA (https://www.jax.org/strain/007004), TITL-GCaMP6s; Ai94 (https://www.jax.org/strain/024104), tetO-G6s (https://www.jax.org/strain/024742), TLX3-Cre (https://www.mmrrc.org/catalog/sds.php?mmrrc_id=41158) and Grik4-Cre (https://www.jax.org/strain/006474)
  ! CAUTION All animal experiments must comply with the relevant institutional and national animal care guidelines. All experimental procedures were carried out under Project Licence 70/14018 (PCC4A4ECE) issued by the UK Home Office in accordance with the UK Animals (Scientific Procedures) Act (1986) and were also subject to local ethical review at University College London.
  ▴ CRITICAL We have typically used 4- to 6-week-old mice of both sexes; it is important to consider the sex of the mice when evaluating experimental results^141^. Note that exact animal requirements will vary for different all-optical implementations, but those used in this protocol are listed here.

### Reagents

- Viruses encoding the indicator and/or opsin: AAV1-Syn-GCaMP6s-WPRE-SV40 (https://gtp.med.upenn.edu/; AV-1-PV2822), AAV1-C1V1-Kv2.1-mScarlett (gift from Chris Harvey^19^), AAV1-C1V1-Kv2.1-mRuby (gift from Chris Harvey^19^), AAV8-CaMKII-GCaMP6m-p2a-ChRmine-TS-Kv2.1-HA (https://neuroscience.stanford.edu/research/programs/community-labs/neuroscience-gene-vector-and-virus-core; GVVC-AAV-180) and AAV-Flex-ChroME-p2a-mls-mRuby (gift from Hillel Adesnik^16^)
  ▴ CRITICAL Resources from which to obtain viruses include: https://vvf.ethz.ch/, https://www.addgene.org/viral-service/aav-prep/, https://neuroscience.stanford.edu/research/programs/community-labs/neuroscience-gene-vector-and-virus-core, https://www.med.unc.edu/genetherapy/vectorcore/in-stock-aav-vectors/, https://www.pvm.cnrs.fr/plateau-igmm/, https://vcf.charite.de/en/, https://www.ntnu.edu/kavli/viral-vector-core and https://gtp.med.upenn.edu/.
- If virus needs to be diluted to achieve a suitable concentration for injection, formulation buffer composed of: Tris (Sigma-Aldrich, cat. no. 77-86-1), NaCl (Sigma-Aldrich, cat. no. 7647-14-5) and Pluronic F-68 (Sigma-Aldrich, cat. no. 9003-11-6)
- If anesthesia is required: anesthetic (e.g., isoflurane; National Veterinary Service, cat. no. 115095) and eye lubricant (e.g., Allergen Lacri-Lube; Allergen Pharmaceuticals)
- Immersion fluid if using immersion objectives (e.g., distilled H_2_O)
- Sucrose water for behavioral experiments

### Equipment

- Surgery setup requires a pipette puller and beveller, an injection syringe pump with stereotaxic control and a microscope. The animal is anesthetized and held in place by ear bars before implantation of a headplate. For preparations involving deep brain areas, a cortical aspiration setup may be necessary.
- Headplate (custom built)
- Chronic optical window (with a cannula for deep preparations)
- Two-photon all-optical microscope for in vivo imaging and SLM-based photostimulation (Bruker, Thorlabs, Scientifica, 3i, custom build, etc.)
- Objective suitable for two-photon imaging (e.g., Nikon 16×/0.8-NA, Leica 25×/0.95-NA, Thorlabs 10×/0.5-NA)
- Imaging laser suitable for two-photon excitation of the indicator and opsin-fluorophores (e.g., a tunable Ti:sapphire high-repetition-rate laser such as a Coherent Chameleon or a SpectraPhysics MaiTai capable of 920 nm and 765 nm for our combination of GCaMP6s and C1V1-Kv2.1-mRuby, respectively)
- Photostimulation laser suitable for two-photon activation of opsins (e.g., Amplitude Satsuma, Coherent Monaco, Menlo BlueCut and SpectraPhysics Spirit)
- Fluorescent plastic slide for calibration routines (Chroma; Thorlabs) ! CAUTION Two-photon lasers used for photostimulation tend to have very high average powers (>5 W) and pulse energies (>10 µJ) and can cause serious burns and eye damage as well as damage equipment. Always comply with laser safety regulations and ensure that all elements in the photostimulation light path have the requisite optical power tolerance.
- Microscope control software (PrairieView for Bruker systems, ThorImage for Thorlabs systems and ScanImage for Scientifica systems)
- MATLAB (>2016a) for custom calibration, setup and analysis programs
- Custom calibration, setup and analysis programs (e.g., Naparm control software (https://github.com/llerussell/Naparm), STAMovieMaker software (https://github.com/llerussell/STAMovieMaker), TransformMaker (https://github.com/llerussell/SLMTransformMaker3D), PhaseMaskMaker (https://github.com/llerussell/SLMPhaseMaskMaker3D) and RawDataStream (https://github.com/llerussell/Bruker_PrairieLink)
- Head-fixation apparatus (custom built; for an example, see ref. ^142^)
- Animal-holding platform/treadmill (custom built; for examples, see refs. ^40,142^)
- Soundproof enclosure for controlled behavioral experiments (custom built). Generally, this will be a cuboidal box with walls of a rigid panel exterior layer and a soundproof foam interior layer (~5 cm thick), with an inner cavity of sufficient dimensions to house all necessary behavioural experiment apparatus (in our case, ~1 m^3^). One wall must be removable/openable to allow animals to be transferred to/from the box, and hole(s) may need to be drilled at strategic points to allow any necessary electrical cabling/reward delivery tubing into/out of the box.
- Behavioral control software and hardware (e.g., PyBehaviour (https://github.com/llerussell/PyBehaviour)) with lick and motion detectors, stimuli presenters and reward delivery
- Software for embedding two-photon optogenetic stimulation patterns into behavioral paradigms (e.g., Two-Photon Behaviour Sequencer (TPBS; https://github.com/hwpdalgleish/TPBS))
- Data acquisition cards (e.g., National Instruments) and synchronization software (PackIO for most systems (http://www.packio.org) or ThorSync for Thorlabs systems)

### Reagent setup

1. The formulation buffer (if virus dilution is required) consists of 20 mM Tris, 140 mM NaCl and 0.001% (vol/vol) Pluronic F-68 (pH 8.0). Once made up, this can be stored in a refrigerator (~5 °C) for ≤1 month.

## Procedure

### Procedure 1: calibration of the all-optical system • Timing 3 h

▴ CRITICAL A critical step in successful all-optical experiments is achieving accurate targeting of the two-photon photostimulation laser to neurons identified with two-photon imaging (Fig. 4a). The first step is ensuring that the two laser paths are physically co-aligned as much as possible by adjusting mirrors to hit established alignment targets. Minor corrections to the co-registration can be made by applying an offset to the galvanometer mirrors specific to the photostimulation pathway, ensuring that both beams point to the center of the FOV. However, because the two optical pathways are independent, they still operate in different coordinate systems. When using an SLM, the mapping from SLM-space coordinates into imaging coordinates needs to be computed. To calculate this required transformation, before the actual experiment, we focus the photostimulation laser into arbitrary spot patterns to burn holes in a fluorescent plastic slide (Chroma; Thorlabs); then, by imaging the same area with the imaging laser, we can register the intended (programmed) locations of the burns with the actual achieved location of the burns in imaging space (Fig. 4b–d). Note that the burnt spots are not necessarily parfocal with the imaging plane, and therefore an imaging z-stack of the plastic slide is acquired. This registration is well captured by an affine transformation (a geometric transformation that preserves collinearity) between the two coordinate systems and results in a mapping from SLM coordinates to imaging coordinates, enabling us to program the SLM to target identified neurons in the imaging FOV (Fig. 4e,f). Co-alignment (Procedure 1, Steps 12–14 below) should be checked often to ensure accurate targeting in all experiments. We recommend checking this often (e.g., daily) in the first instance to confirm that there are no drifts in the system but eventually less often (e.g., weekly to monthly) if there is sufficient temperature stability in the room and pointing stability of the laser source, both of which are required for consistent optical alignment.

▴ CRITICAL The procedure to align and calibrate the two light-paths is given in Procedure 1. Procedure 1 would not need to be carried out before every animal’s surgery, only once when you build the system (and then maybe at future time points if the system begins to lose calibration).

1. Center galvanometers in both imaging and photostimulation pathways.
2. Ensure adequate physical alignment of the photostimulation beam through the entire pathway up until the SLM, hitting alignment targets at various manufacturer design points. The beam should overfill the active surface of the SLM to maximize optical resolution, especially where power throughput is not a major concern. Similarly, ensure adequate physical alignment of the imaging beam through the entire imaging light path up until the back aperture of the objective.
3. Unblock the zero order of the SLM by translating the block (see above) out of the way and continue to optically align through the system with this beam, again hitting the manufacturer alignment points, culminating at the back aperture of the objective. Both beams should hit the center of the back aperture. Depending on the magnification factor, the photostimulation beam may be smaller than the imaging beam, and the appearance of the rectangular reflective surface of the SLM may be visible. Note that the coarse global alignment of the photostimulation and imaging light-paths described in this step precedes, and is distinct from, the fine-scale alignment of photostimulation galvanometer coordinates to imaging galvanometer coordinates via an empirically calculated transform described later in Procedure 1, Step 10.
4. The SLM’s efficiency is in part dictated by the polarization of the laser beam. The polarization of the beam can be adjusted with a half wave plate on the optical table. One way to do this is as follows. Position a fluorescent card at the zero-order block position to visualize the diffraction pattern. Apply an arbitrary diffraction pattern and optimize the power distribution into the first order spots, and out of the zero-order spot, by rotating the half wave plate. After optimization, remove the fluorescent card.
  ▴ CRITICAL STEP Other methods to more accurately optimize the polarization could be to focus the SLM spot pattern on a fluorescent slide under the objective and visualize the pattern with a camera and repeat the procedure by using pixel intensity to quantify the ratio of zero-order to first-order brightness. Another method would be to position a power meter under the objective—after having blocked the zero order—and optimize the polarization to reach maximum power intensity, which corresponds to the optimum first-order diffraction.
5. The SLM’s efficiency is also dictated by the lookup table by which hologram pixel values are converted to voltages on the SLM itself. It is usually provided by the manufacturer (but can also be calibrated manually). Set this lookup table in the software driving the SLM in order for it to function optimally.
6. Now that the photostimulation beam is physically coaligned with the imaging pathway, position a plastic slide under the objective.
7. Ensure parfocality of the imaging and photostimulation beams. Upload a phase mask to the SLM (generated from a known target pattern via the Gerchberg-Saxton algorithm^132^; see Experimental design, Patterned illumination device for more details) and burn the spot pattern in the plastic slide. Find the axial center of the burn location by moving the imaging z-focus. One way to fine-tune the parfocality is to make slight adjustments to the vergence of the stimulation beam (assuming the imaging pathway is well collimated) by adjusting the second lens of the post-SLM telescope to fine-tune the parfocality until the burn location is at the nominal imaging plane.
  • Burn parameters: power of 50 mW (28 mW/µm^2^) per spot, 10-ms duration, repeated until burns are visible (Amplitude Satsuma 20 W, 2 MHz)
8. Remove the phase mask on the SLM by uploading a blank phase mask, so that only the zero-order beam is propagated through the system.
9. Decide on the optical zoom of the imaging pathway to be used for experiments, because the photostimulation calibration is specific to particular imaging conditions.
10. Perform the manufacturer’s procedure to align the galvanometer pointing of the photostimulation pathway (using the unblocked zero-order beam) with the imaging pathway. This step generally consists of an empirical calculation of a transform (e.g., an affine transformation) between photostimulation and imaging galvanometer coordinates and is distinct from the global light-path alignment procedure in Procedure 1, Steps 3 and 6.
  - Burn parameters: power of 50 mW (28 mW/µm^2^), 10-ms duration, repeated until burns are visible (Amplitude Satsuma 20 W, 2 MHz)
11. Now, re-block the zero order by translating the block into place. This is straightforward to achieve when using a camera to visualize a diffraction pattern (e.g., a grid of spots) on a fluorescent plastic slide, because the zero-order block will occlude a region of the pattern.
12. With the zero-order block in place, map out the region that is blocked and thus inaccessible. Again, using a grid-like spot pattern and visualizing the fluorescence with a camera, we can calculate the size of the block in physical space by recording which points of the grid are not visible. This region of SLM space is essentially unaddressable, but note that by translating the galvanometer pointing position, the blocked region can be circumvented. This is because the location at the sample plane of the unaddressable zero-order beam blocked region is dictated by the angle of the photostimulation galvanometers (if present); thus, if this region contains neurons that one wants to target, one can use the photostimulation galvanometers to ‘point’ the unaddressable zero-order block region elsewhere and use the SLM to steer beamlets to target the neurons of interest (see Fig. 8a,b for detail).
13. Next, perform the SLM targeting calibration. Display an arbitrary, rotationally asymmetric pattern (with no transform applied) on the SLM and burn spots in the plastic slide. If performing a 3D calibration, burn this pattern at various axial offsets by using 3D phase masks. The arbitrary SLM Z-coordinate range can be found by trial and error until the burns are within the desired volumetric imaging range. Take an image (or a stack if performing a 3D calibration).
  - Burn parameters: power of 50 mW (28 mW/µm^2^) per spot, 10-ms duration, repeated until burns are visible (Amplitude Satsuma 20 W, 2 MHz)
14. Inspect the acquired image and record the coordinates of the burn locations in the image and use these to compute the affine transformation between the intended (i.e., programmed SLM coordinates) and the actual burn coordinates. See SLMTransformMaker.m for an example implementation.
15. Generate new spot patterns—with the newly calibrated transform applied—and again burn spots in the slide. Take an image (or 3D stack) and calculate the distance from intended targets to the actual burns to test the accuracy of the calibration. Redo Procedure 1, Steps 12 and 13 if necessary (e.g., if the burnt spots are greater than 2 µm from the intended targets (acceptable accuracy will depend on the structures being targeted)).
16. Finally, calibrate the total power throughput (i.e., the average power on the sample) to ensure safe and effective stimulation of neurons. Apply a typical SLM spot pattern (i.e., one similar to what might be used during an experiment) and measure power on the sample after the objective with galvanometers centered. Record the power at intervals of the power-modulation device setting. This number is used later in combination with the number of intended neuron targets to set the total power level (split between all the targets).
  ▴ CRITICAL STEP (Optional) To calibrate the reduction in efficiency with larger diffraction angles, displace a single point to increasing offsets from the zero order and record the power at each location. The relationship between distance and power can be used to scale the power distribution among spots to equalize the power delivered among spatially dispersed neurons in a group. These weights can then be implemented in a weighted phase mask generated by the weighted Gerchberg–Saxton algorithm^132,143^.

### Procedure 2: surgery • Timing 3 h

! CAUTION Ensure that relevant regulations and guidelines for sterile recovery surgeries are followed at all times.

▴ CRITICAL Optical access must be gained for the brain area(s) of interest by surgery, following Procedure 2, for each animal used on a given calibrated system, and the target neurons must be engineered to express two proteins, an opsin and an indicator.

1. Determine the best expression strategy for the desired experimental protocol (Fig. 5a and Experimental design, Choice of indicator/opsin combination), taking into consideration viral injection volumes and concentrations if using virally expressed constructs, or the complexity of combining genotypes if using transgenics.
  ▴ CRITICAL STEP Adeno-associated virus (AAV) serotypes and promoters should be chosen to ensure specificity of expression in the cell population of interest while also allowing sufficient overlap of opsin and indicator expression (see Supplementary Table 1 for details of key published combinations and Procedure 3a, Step 4 for more detail on checking expression on your own system).
2. Because all experiments will require optical access to the brain ROI, ensure that the optical setup of the microscope, surgical preparation and spectral properties of all constructs are appropriate, particularly when the brain ROI is deep (Fig. 5c,d). Possible optimizations could be to use far red–shifted indicators and/or opsins to reduce scattering of excitation photons, or methods to improve access to deep structures (i.e., GRIN lenses and cortical aspiration).
3. Decide on surgery protocol, by using appropriate coordinates, to target your brain area of interest (Fig. 5b–d) and perform the surgeries^14,20,40,41,43,144^. Express constructs, either through stereotactic viral injection into the brain area of interest^145^ or via endogenous expression in transgenic mice^146,147^. To provide optical access to expressing brain tissue replace the overlying skull with a chronic window^144^, and for deep structures, remove overlying brain tissue^140^. Install a headplate to enable head fixation under microscopes^142^.
  ! CAUTION Allow sufficient recovery time between surgery and subsequent procedures according to the guidelines of your institution (typically 2–7 d and/or once animals have returned to presurgery weight).
  ▴ CRITICAL STEP Ensure that there is sufficient spectral separation between the two-photon excitation spectra of opsin and indicator to avoid cross-talk in either direction. The imaging laser wavelength should not significantly increase spiking in opsin-expressing neurons^14^; nor should the photostimulation laser wavelength induce appreciable fluorescence of the indicator (although note that photostimulation lasers may also cause significant, and unavoidable, autofluorescence of endogenous fluorophores in the tissue). In practice, photostimulation can cause imaging artefacts. However, because these are limited to the exact time of photostimulation and because most calcium indicators have a slow decay, this may not be an issue in practice if photostimulation epochs can be discarded and responses can be analyzed in the poststimulus window.
  ▴ CRITICAL STEP Ensure that the expression strategy yields robust expression in sufficient numbers of neurons for your experimental purposes without damaging the ROI (see Supplementary Table 1 for some key published combinations). If using viral strategies, make sure that the total volume of virus injected and the number and proximity of injection penetrations to the site of interest do not damage surrounding tissue. If using transgenic strategies, which tend to drive expression in fewer neurons than virally mediated expression, make sure that any transgenic lines used yield sufficiently dense expression (see Procedure 3a, Step 4 and associated Critical step for more detail).
? TROUBLESHOOTING
4. If using viruses, allow sufficient time for constructs to express to useable levels (~2–3 weeks for AAVs^148^). For virally expressed indicators such as GCaMP, the window of useable expression is generally thought to be ~3–15 weeks^23,24^, although this depends on the concentration of injected virus^14^.
  ! CAUTION Over this period, increasing proportions of neurons transition from a ‘donut-shaped’ annulus (with indicator fluorescence in the cytoplasm surrounding the dark, indicator-free nucleus) with low baseline fluorescence and high signal dynamic range to a homogeneously fluorescent body (as the nucleus itself fills with indicator) with high baseline and low signal dynamic range^23,24^. This is a continuous process that can progress at significantly different rates in different animals, and in different regions within an animal, for reasons that have not been fully characterized (although this probably stems from unavoidable inconsistencies in viral volume injected, tissue damage caused by injection and inhomogeneities in viral concentration as virus spreads through the tissue away from the injection site). Note that some indicators such as YC3.60^100^ and the bicistronic mRuby2-GCaMP family^103^ potentially show more stable expression. For virally expressed opsins, high titers are typically used to maximize the sensitivity of neurons to light (and therefore minimize light-induced damage to tissue) with minimal adverse effects on neuronal health and function^27,120,126,149^, although presumably extreme overexpression of opsin could be associated both with the general cytotoxic effects of overexertion of cellular protein production machinery and with the specific effects of inserting additional exogenous membrane conductances (changes in membrane resistance, resting potential, propensity to spike and spike characteristics). If using transgenics to drive expression, ensure that surgery and any subsequent experiments are done during the period in the mouse’s life cycle during which the transgene of interest is robustly expressed. As far as we are aware, the only major deleterious effects of transgenic expression of calcium indicators reported so far is the aberrant, epileptiform cortical activity and associated behavioral seizures exhibited by some transgenic GCaMP indicator mouse lines^90^. Fortunately, given that these lines incorporate a tetracycline transactivator (tTA) regulatory element, this can be avoided by giving doxycycline in the drinking water from birth until 7 weeks (to block transgene expression during the problematic developmental period), at which point doxycycline can be removed to allow normal expression to ramp up within 3–4 weeks (with no neural/behavioral issues)^90^. It should also be noted that there are transgenic GCaMP lines without such issues^150^. For all methods of expressing all-optical constructs, it is advisable to pilot experiments with previously reported volumes and concentrations and to assess and continuously monitor expression during subsequent experiments. See Procedure 3a below for details on identifying usable expression.

### Procedure 3a: visualizing opsin and indicator expression • Timing 10–20 min per mouse

1. After having allowed animals to express constructs for a sufficient time (see Procedure 2), head-fix the animal beneath the microscope^142^. If anesthesia is required (e.g., if animals have not yet been habituated to head fixation, or if subsequent sensory stimulus mapping requires mice to be anesthetized), then first anesthetize in 5% (vol/vol) isoflurane in an induction chamber and then transfer to the two-photon microscope, maintaining anesthesia with 1% (vol/vol) isoflurane and mouse body temperature with a heating pad/blanket.
  ! CAUTION All animal experiments must comply with the relevant institutional and national animal care guidelines.
2. Navigate to the relevant FOV.
3. Take anatomical image(s) of plane(s) of interest to check expression of opsin (e.g., at 765 nm for C1V1-Kv2.1-mRuby) and indicator (e.g., at 920 nm for GCaMP6s) (Fig. 5b–d).
4. Ensure that expression is sufficient for your experimental purposes (for examples of our setup, see Fig. 5; for your own setup, please follow calibration guidelines in Experimental design and see the final Caution in Procedure 2). Typically, this should be as robust as possible (not underexpressing) without causing neuronal damage (not overexpressing). Specifically, cytosol-filling indicators (such as GCaMP) should show low baseline fluorescence and high SNR transients with few neurons having filled nuclei (<10% of neurons^14,23,24^). Opsin-conjugated fluorophores (if present) should be clearly visible in appropriate cell compartments (i.e., only in the soma if using soma-restricted opsins) with reasonable laser power (<50 mW) (Fig. 5b–d), and any two-photon photostimulation should yield reliable calcium transients (see Procedure 4b, final Critical step below for details) in targeted neurons without excessive recruitment of off-target neurons (i.e., significant activation of neurons outside the measured resolution of the system that decays with distance from stimulation sites).
  ▴ CRITICAL STEP Ensure that both opsin and indicator express in enough neurons in the neural population of interest and that there is sufficient overlap between them. The requisite number of construct-expressing neurons will vary, but for our experiments using all-optical techniques to modulate behavior, we typically use FOVs in which we have identified >150 opsin-expressing neurons and >500 indicator-expressing neurons in a given imaging plane (710 × 710 µm *XY* dimensions) of our four-plane volumetric stack (33-µm spacing; 100-µm axial extent) ~150–250 µm below the pia in the L2/3 S1 barrel cortex^40^, corresponding to a pool of >600 opsin- and >2,000 indicator-expressing neurons across the volume. Ideally, overlap between opsin and indicator would be 100%, as it is when using bicistronic opsin-indicator constructs^37^. However, in our experiments using separate viral opsin and indicator constructs, or viral opsin in indicator transgenic mice, we routinely use FOVs in which only 40–50% of indicator-expressing ROIs also have opsin, corresponding to >50 dual-expressing neurons in a given FOV as described above^40^ and therefore >200 total dual-expressing neurons across the volume to choose from for all-optical interrogation.
  ▴ CRITICAL STEP Always use similar laser power for expression checking (30–50 mW for cortical depths of 100–300 µm) to ensure that differences in apparent expression clarity/brightness are due to the expression itself and not variation in strength of fluorophore excitation.
? TROUBLESHOOTING

### Procedure 3b: training animals on a behavioral task • Timing 7–10 d

▴ CRITICAL To probe the neural basis of a perceptual or behavioral function, we require the animals to perform a reliable and repeatable behavior. Typically, these are tasks whereby the animal indicates the presence of a particular stimulus, in most cases by licking at a water spout to receive a sugar water reward for the correct response. Mice are motivated to perform the tasks by being placed on a food or water restriction diet and learn the task through a series of iterative steps^142^. The time taken for animals to reach good performance on the task will depend on the complexity of the task.

1. Train animals on the desired behavioral task, following the normal habituation, learning and training phases (Fig. 6; see the two Critical step below). Note that a period of habituation to handling and head fixation before training is highly recommended to minimize animal stress and maximize good behavioral performance^142^.
  ! CAUTION Using laser shutters on the photostimulation light-path can cause perceptible noises coincident with photostimulation epochs. This should be taken into account when designing behavioral experiments. Some options to alleviate this are to mask the shutter noise with sound-insulating material and/or constant white noise or to use non-mechanical power-modulation devices (a Pockels cell or acousto-optic modulator).
  ▴ CRITICAL STEP Choose an appropriate behavioral task design^151^ for your experimental question, because this strongly dictates the types of all-optical manipulations possible and the causal inferences that can be drawn. For instance, in our laboratory, we routinely use the following: (i) detection tasks to assess how the number and functional identity of stimulated V1 neurons influence the detection threshold of mice trained to detect oriented gratings (Fig. 6a), (ii) discrimination tasks to assess how much additional activity in S1 is required to bias animals to choose stimulation of one whisker over another (Fig. 6b) and how this is integrated into local network processing of sensory information and (iii) complex behavioral tasks, such as head-fixed navigation along virtual linear tracks, to compare how targeted place cell perturbations influence local network activity in hippocampal CA1 and CA3 (Fig. 6c).
  ▴ CRITICAL STEP Ensure that the task can be learned in a timeframe such that the time when animals perform at desired levels coincides with the period during which constructs are optimally expressed (i.e., if using virally mediated constructs, before constructs begin to overexpress and degrade cell health; if using transgenic animals, then after transgene expression has begun and before it ceases; see Procedure 2, Step 4, final Critical step and final Caution for detail). If this presents a problem, consider a two-stage surgical protocol in which initial installation of a head-fixation device allows prior training to the desired level of performance before subsequent expression of constructs and chronic window installation.

### Procedure 4a: mapping functional properties of neurons • Timing 1–2 h per FOV per mouse

1. Head-fix the animal beneath a two-photon microscope. If anesthesia is required, then first anesthetize in 5% (vol/vol) isoflurane in an induction chamber. Then, transfer to the two-photon microscope, maintaining anesthesia with 1% (vol/vol) isoflurane and mouse body temperature with a heating pad/blanket.
  ! CAUTION All animal experiments must comply with the relevant institutional and national animal care guidelines.
2. Find and map the FOV in your brain ROI (Fig. 7). An appropriate protocol for this will be similar to most functional mapping experiments used in correlational studies, using both widefield coarse resolution and two-photon cellular resolution mapping to first identify the general brain area and then specific two-photon imaging FOV(s) of interest^150,152–154^; however, it should be optimized to allow fast online analysis (Fig. 7a; see Critical step below). We briefly present an example below (Procedure 4a, Steps 3–6 and Fig. 7b–j).
  ▴ CRITICAL STEP Online analysis allows functional mapping to inform subsequent all-optical interrogation even within the same experimental session without the animal becoming too unmotivated and/or tired to perform. The major optimization that we have found useful is to motion-correct our two-photon time series in real time by streaming raw pixel data from the microscope acquisition software directly through a custom pipeline in MATLAB (RawDataStream; https://github.com/llerussell/Bruker_PrairieLink). This reduces post-acquisition time to motion-correct the data from ~1 min per minute of acquired data per plane to essentially zero. By writing the data to a raw binary file, readable by any programming language, we avoid the TIFF file format and the overheads associated with slow loading. After the data are acquired, we use this already-motion-corrected data to identify stimulation targets in one of two ways. First, we generate pixel-wise STA images (by using STAMoviemaker; https://github.com/llerussell/STAMovieMaker) in which each pixel of the resulting image conveys the following information about the trial-averaged poststimulus response of that pixel: preferred stimulus identity (hue), preferred stimulus tuning strength (saturation) and preferred stimulus response amplitude (value, or brightness). This gives a quick, intuitive map of the strength and tuning of functional responses that is automatically in register with the spatial position of the imaged neurons, providing the information needed to place photostimulation targets at the spatial location of functionally tuned neurons of interest (see below and Fig. 7b,c,e,f,h,j for examples). Alternatively, when more sophisticated analysis of responses is required, we use a version of the Suite2p toolbox^78^ that we have modified to have five main optimizations for real-time analysis of all-optical experimental data: (i) it imports raw real-time, motion-corrected, time-series binary files (instead of un-motion-corrected TIFFs) and can therefore skip the motion correction step; (ii) it optionally imports a file recording the correlation of each acquired frame with the reference image used for real-time motion correction (simultaneously recorded during real-time motion correction; see above), which can be used to identify and exclude imaging frames corrupted by photostimulation artefacts (which typically have low correlation with the reference image); (iii) if multiple planes are imaged, then analysis is parallelized on the CPU to process as many planes simultaneously as possible; (iv) data are imported as memory-mapped files to speed up data manipulation during ROI segmentation and trace-extraction phases; (v) the ROI segmentation procedure is terminated after a fixed number of iterations that is manually selected to yield a balance between speed and accuracy with the data acquired on our system. These modifications allow users to skip the lengthy motion-correction step, quickly process multiple planes and generate ROIs and traces in ~5 min/plane (plus 5 min for manual ROI curation) (https://github.com/hwpdalgleish/Suite2P-master_online). This allows us to confirm the intuitive results of STA image analysis and generate target groups on the basis of statistical comparisons between neuronal traces.
  ! CAUTION Pay attention to the shifts applied to motion-correct the image with respect to the reference image. A few-microns shift is tolerable, but larger shifts will require the FOV to be repositioned to ensure high photostimulation efficiency when neurons are targeted with the stimulation laser. Note that this can be done online, by displaying a continuously updating record of the XY shift between each incoming frame and the reference image and using this to update the objective position relative to the FOV if these shifts become too large, either manually or programmatically.
3. Set up stimuli to map, e.g., visual gratings for V1 (Fig. 7b–d), whisker vibrations for S1 (Fig. 7e–g) or navigation in virtual environments for hippocampal CA1 and CA3 (Fig. 7h–j).
4. Perform widefield calcium imaging during delivery of a series of repeats (~10) of the desired stimuli and generate STA images (e.g., by using STAMovieMaker; see the above Critical step; Fig. 7b,e).
5. To find a two-photon FOV for all-optical interrogation that has the functional responses desired and robust, healthy construct expression, use STA images from functional widefield calcium imaging (see the preceding step and the Critical step that precedes it; Fig. 7b,e) and structural construct expression images (see Procedure 3a; Fig. 5b–d).
6. Map the functional responses of the desired FOV at the cellular level by using two-photon imaging, generate two-photon STA images (Fig. 7c,f,h,i) and use online ROI/trace analysis to extract trial-wise traces (Fig. 7d,g,j; left), which can be used to extract average functional tunings (Fig. 7d,g,j; right).
? TROUBLESHOOTING

### Procedure 4b: mapping the photostimulation response of targeted neurons • Timing 30 min per FOV per mouse

▴ CRITICAL Before performing all-optical experiments, it is beneficial to know which neurons are photostimulatable (i.e., identify neurons that express both the indicator and opsin to sufficient levels to enable optogenetic activation while their activity is recorded). Baseline marker fluorescence is insufficient to ensure functional expression levels. Therefore, to identify these cells, we photostimulate each and every cell in the FOV and record their responses. This could be achieved by stimulating single cells, one by one, but this becomes time consuming when the number of cells in the FOV is high. A second option would be to photostimulate all cells at once, but this is appropriate only if the number of cells in the FOV is low (because of a limited laser power budget or to mitigate concerns over heating). The optimal solution is to group all the neurons into a number of groups of a defined size and stimulate the groups one by one. We have designed a piece of software (Naparm) that implements this stimulate-all-cells ‘mapping’ protocol and is flexible enough to implement any other type of all-optical experiment in which only a certain group or groups of selected neurons are stimulated. This software makes it intuitive to identify ROIs in an FOV and design stimulation group and parameters and then generate all the files required to configure the microscope to execute the experiment.

! CAUTION Stimulation protocols used to identify and map neurons expressing opsin may induce plasticity and generation of artificial ensembles^36,44^. To avoid this issue, care should be taken to limit the number of photostimulation repeats used in this step to a number sufficient to obtain statistically reliable estimates of photostimulus response amplitude.

1. Run Naparm and import relevant FOV image(s) (either those acquired in Procedure 4a if your microscope is still positioned over or can navigate back to exactly that FOV or those newly acquired during this procedure) by either dragging images into the image window (left) or clicking the *Load image(s)* button in the Image panel. For standard photostimulation response mapping, this will likely be the opsin image(s). Note that multiple image(s) of the same plane(s) can be imported in this way and viewed via the drop-down menu in the Image panel.
2. Select opsin-expressing neurons to photostimulate. Either do this manually by left-clicking on cells in the image window (left) or use one of the automatic detection options in the *Automatic* section of the ‘Add points’ panel.
  ▴ CRITICAL STEP (Optional) Note that by applying a grid of equally spaced points over the whole FOV in lieu of precisely targeted neurons, a good estimate of neuronal photostimulation responses can still be obtained without the time-consuming step of identifying hundreds (or thousands) of neurons (Fig. 8i).
3. Once all neurons have been selected that are relevant to your experimental goal (e.g., neurons tuned to a particular visual grating orientation if you are testing the impact of ensemble co-tuning via all-optical interrogation during a visual discrimination task), group them into stimulation groups (cells within a group will be stimulated simultaneously, and groups will be stimulated sequentially). Select the desired grouping method from the ‘Assign groups’ panel and adjust the *Group size* or *Number of groups* options to the desired value. For standard photostimulation response mapping, we use the *ekmeans* algorithm to group neurons into spatially clustered groups of equal size. Click *Group* to group cells and note that selected cells in the image window are now colored by group and associated with the centroid of all cells within their group. For randomly seeded algorithms (*ekmeans* and *Random*), clicking *Group* multiple times will repeat the grouping procedure and reassign cells to groups. In general, we use a group size of 50, grouped via the *ekmeans* algorithm.
4. Set up the timing structure of how and when groups are stimulated within a single trial of the protocol; this will define the stimulation pattern both for a given group and the order in which the groups are sequenced. In the ‘Single trial’ panel, for a single group, set the number of times to stimulate it, at what rate and with what photostimulus duration with the *Shots per pattern, Inter shot interval (ms)* (i.e., the timing between spiral onsets) and *Spiral duration (ms)* fields, respectively. Use the *Delay first spiral (ms)* to define the time it takes for your SLM to update to a new pattern. For our system using a BNS P512-1064 SLM, we use 5 ms (for our system using a Meadowlark P1920 SLM, we use 20 ms because of slower pixel response times). Define the time between each group with the *Change pattern every (ms)* field. The *Trigger each pattern* checkbox enables you to decide whether to trigger each pattern individually (checked) or just the first pattern (unchecked). Note that the latter option assumes that the sequence of patterns will be generated by some external software (e.g., the microscope software itself (as in the Bruker system)). The entire sequence of groups can then be repeated on a given trial by setting the *Sequence repetitions* and *Sequence repetition interval (ms)* fields. Note that all these fields will dynamically update the trigger display below. Colors in this display correspond to group colors in the image window (left). In general, we stimulate 50 cell patterns (the total number of patterns depends on the total number of targets in the FOV) with 10 shots at 20 Hz (50-ms inter-shot intervals) with a spiral duration of 20 ms and a 5-ms delayed first spiral. We sequence from one pattern to the next every 1 s.
5. Set up the timing of how many and how often single trials are delivered in the complete protocol. In the ‘All trials’ panel, update *Number of trials* to define the number of repeats of the single trial defined above. Set the inter-trial interval with the *Trial length (s)* field. Note that this is inclusive of the time it takes for the sequence of stimuli to be stimulated. If appropriate, add a period of spontaneous imaging (i.e., no photostimulations) before and/or after the photostimulation mapping period. Again, note that changing these fields will dynamically update the trial triggers display below. In general, we do 10 trials, separated by a minimum of a 10-s inter-trial interval.
6. Set the parameters of how the neurons are stimulated by the laser, in this case, in terms of power and spiral shape/size. In the ‘Spiral parameters’ panel, the *Revolutions* field defines the number of revolutions that describe the spiral shape itself. To repeat a given spiral multiple times, change the *Shots per pattern* field in the ‘Single trial’ panel. The *Laser power* field defines the power distributed across all targets in a given group. In general, we use three spiral revolutions, a 15-µm spiral and a laser power that provides 6 mW (3 mW/µm^2^) per cell.
  ! CAUTION Ensure that your laser power is calibrated (see Procedure 1, Step 16) so that the value entered into Naparm corresponds to the power on the sample. Also make sure to test safe laser powers per cell for your desired photostimulation pattern and be careful not to exceed this (Box 2).
7. Depending on the FOV size (imaging and stimulation), dispersion of the neurons within and between the groups and the diffraction efficiency of the SLM, decide whether to use galvanometer/SLM (whereby the galvanometers are steered to the centroid of each group and the SLM patterns are relative to this new set point) or pure SLM targeting with the *Centroids* and *All points* buttons, respectively, in the ‘Mark Points mode’ panel. Note that the SLM-to-imaging transform calculated in Procedure 1 (with photostimulation galvanometers centered) will be valid at all galvanometer offset locations, but ensure that you take account of the global galvanometer offset when calculating phase masks to be displayed on the SLM.
8. Now that the experiment is designed, export all necessary files (see below) to then load them into and configure the microscope software. In Naparm, export these files by using the *Export all* button at the bottom right of the graphical user interface (GUI). This will output a folder with a user-defined name into a user-defined path (the path and name are defined earlier by the ‘…’ button and text box in the ‘Save path’ panel) containing the requisite files for the microscope system. These files should be loaded into the relevant sections of the microscope control software.
  - This folder will contain a file with the photostimulation galvanometer locations (.gpl file for Bruker systems; .bmp for Thorlabs systems), a .xml file and a folder of phase masks. The .xml file defines the photostimulation protocol (timing, spiral parameters and photostimulation power).
  - If using external software to upload the phase masks to the SLM (i.e., not driven by the microscope software), then the folder of phase masks will contain all phase masks used to complete a single repetition within a single trial. Load this folder of phase masks into your SLM control software (in our case, Blink with OverDrive Plus) and set the number of repetitions to a value appropriate for your protocol.
  - The .dat files contain triggers for the SLM updates (changing patterns) and spiral delivery. These should be loaded into the master synchronization software (in our case, this is PackIO).
9. Set up an imaging time series (t-series) acquisition of the plane(s) of interest with the requisite number of frames. Allow a buffer of ~10 s before and after the photostimulation mapping protocol for pre- and post-photostimulation analysis.
10. Check via an imaging ‘live scan’ (i.e., not the full acquisition) that the imaging FOV has not moved significantly (laterally and/or axially) in the time it took to set up the photostimulation mapping protocol. Note that some body movements (including licking) can cause small FOV movements in some preparations^155^, so take this into account before physically translating the microscope. To confirm that the FOV has not shifted, we recommend finding an obvious landmark (e.g., a cell highly expressing the indicator) in your anatomical image(s), noting the pixel location of that cell’s centroid and ensuring that the cell is at that location in the live imaging window. By comparing the relative size of the landmark and relative positions of other landmarks in the FOV, the same axial focus can also be confirmed. If using a water immersion objective, also confirm that the immersion fluid level is sufficient. Stop the ‘live scan’.
  ! CAUTION If the FOV has moved >5 µm, then photostimulation targets will no longer efficiently target the desired cells.
11. Begin the photostimulation experiment. Arm all relevant software to wait for triggers. Begin the master synchronization software recording. Begin the t-series. Wait 10 s and then begin delivering the photostimulation triggers from the synchronization software.
12. Once imaging is complete, analyze the results of the photostimulation experiment. One method to do this is via the construction of pixelwise STA images, revealing which neurons were successfully stimulated (Fig. 8g,i) (note that another method is to use extracted traces, and this is described in the Critical step below). To do so, import both the t-series file and synchronization file into STAMovieMaker. This will process and save STA movies and images for further analysis and cell selection. Configure the GUI for photostimulation STA analysis. Update the *Different stims* field in the ‘Stim setup’ panel to the number of groups stimulated. Click ‘Run’ and then inspect the output images and movies for brightly colored (successfully stimulated) neurons.
  ▴ CRITICAL STEP When analyzing extracted traces from imaging data, neurons responsive to photostimulation should show reliable calcium transients (transients on >50% of photostimulation trials) of an amplitude appropriate for your chosen opsin/indicator combination, photostimulation parameters and experimental requirements. For example, for our most commonly used indicator (GCaMP6s), we use the fluorescence change elicited by known numbers of spikes^24^, confirmed with our own simultaneous calcium imaging and cell-attached electrophysiological recordings^14^, to estimate the transient amplitude resulting from a single spike on our system (~0.1 Δ*F*/*F*). We then confirm this for photostimulation with our most commonly used opsin (C1V1) for a given spiral power and duration^14^. Note that one spiral can occasionally elicit multiple spikes^14,19^ with a likelihood that increases with increasing photostimulation power and spiral duration; thus, care should be taken to calibrate photostimulation parameters to ensure that the vast majority of single spirals elicit a single spike^14^. We use this quantal value, in combination with C1V1’s ability to follow photostimulus trains at different frequencies^29^, to estimate the expected transient size resulting from any photostimulus train that we might use. For instance, in most Naparm protocols stimulating neurons 10 times at 20 Hz, given a single spike amplitude of ~0.1 Δ*F*/*F* for GCaMP6s and a 90% response reliability of a given strongly C1V1-expressing neuron to sequential spikes in a 20-Hz train (i.e., firing 9/10 spikes), we would expect a transient of ~0.9 Δ*F*/*F*. In practice, for most of our experiments, we tend to accept neurons that reliably respond less strongly than this expected value (>0.3 Δ*F*/*F* on >50% of trials) because the specific number of action potentials elicited in each neuron is less important to us than the number of neurons that we can activate to any extent. Note also that because response amplitude correlates with opsin expression^29^, not all opsin-expressing neurons will be easily photostimulatable. In our hands, we find that ~60% of GCaMP^+^/C1V1^+^ are reliably photostimulatable in the above manner. The amplitude threshold will therefore be set by the requirements of each experiment. Given this known cell-to-cell variation in sensitivity to photostimulation^16,40,122^, it is possible to tailor the photostimulation power delivered to individual neurons in a targeted population on the basis of their photostimulatability to equalize evoked responses across all neurons stimulated by using an SLM to modulate the intensity of individual diffracted beamlets^16^.
? TROUBLESHOOTING

### Procedure 5: all-optical interrogation during behavior • Timing 3–5 h per FOV per mouse

▴ CRITICAL As described in the introduction, the workflow of the experimental phase of most all-optical behavioral experiments will follow a similar general structure (Figs. 2, 9a). We detail a specific example of this below (Fig. 9b–h). For this experiment, a mouse has been trained over the course of ~5 d to report 1P photostimulation of neurons in the barrel cortex of decreasing power by licking for sucrose at an electronic lickometer. This step is necessary because we have found that naïve animals find it difficult to detect two-photon activation of small numbers of neurons early in training when they are still adapting to head-fixed behavioral training in general^40^. Note that as with repeated two-photon optogenetic activation of groups of neurons^15,36^, there is a possibility that one-photon stimulation protocols may induce plasticity between co-activated neurons, resulting in the generation of artificial ensembles (see Procedure 4b, Caution 1). At the lowest LED power, the mouse is then transitioned to detecting two-photon photostimulation of arbitrary groups of 200 and 100 neurons. In the final phase of the experiment described below, we characterize the functional tuning and photostimulatability of neurons in the C2 barrel (Fig. 9b), identify an ensemble that responds strongly to both whisker stimulation and photostimulation (Fig. 9c,d), embed this ensemble into our behavioral training paradigm as one would any other stimulus type (Fig. 9e) and photostimulate them during the final sessions of this previously learned behavioral task to assess their behavioral salience (Fig. 9f–h).

1. In this example, to identify the C2 barrel of vS1 before the experiment, we anesthetize the animal to facilitate providing sensory stimulation to single whiskers. At least 1 d before the final training sessions, anesthetize and head-fix the animal on a heating pad beneath the microscope (5% (vol/vol) isoflurane for induction; 1% (vol/vol) for maintenance). Note that this step may not be necessary for mapping other sensory modalities, such as retinotopic mapping in V1, which is possible in awake animals.
2. Map the C2 barrel (see Procedure 4a and Fig. 7) by delivering 10 trials of 30-Hz sinusoidal vibration in the dorso-ventral axis with a 1-s stimulus duration and a 10-s inter-stimulus interval by using a piezo electric bender attached to the whisker via a glass capillary while widefield imaging the dorsal surface of the brain through the cranial window by using a blue LED, 5×/0.1-NA air objective and an sCMOS (scientific complementary metal-oxide-semiconductor) camera acquiring at 10 Hz (~1.5 mm × 1.5 mm, 512 × 512 pixel resolution).
3. Use the sCMOS/LED light-path on the microscope to center the objective over the region of indicator/opsin expression visible through the cranial window.
4. Feed the C2 whisker into a pulled glass capillary attached to a piezo bender and turn isoflurane to 0.5% (vol/vol).
5. Acquire a widefield movie while delivering whisker stimuli.
6. To find the best sensory-responsive FOV for subsequent experiments, use STAMovieMaker to create an STA Δ*F*/*F* image of the poststimulus epoch from the widefield movie and stimulus triggers. The peak in this image corresponds to the C2 barrel (Fig. 9b, Widefield sensory mapping: pink region), and the best-expressing FOV near this location will be used going forward (Fig. 9b, Widefield sensory mapping: white dashed box; Fig. 9b, Opsin expression and Indicator expression).
7. Allow the animal to recover from anesthesia (hours to days, depending on the experiment).
8. Immediately before the experimental head-fixation period during which the animal will perform the behavioral task (see the following step), weigh the animal and record its weight. This will be compared with the animal’s weight immediately after it has been removed from the microscope (see Procedure 5, Step 26) for two reasons: (i) to assess general health and (ii) (if your behavioral task requires water/food deprivation) to keep track of how much reward (food/water) the animal consumes during the behavioral training session and to determine how much additional water/food to give the animal after the behavioral training session has finished to allow it to maintain a healthy weight^142^.
9. Head-fix the awake animal on a spherical treadmill under the microscope and use the two-photon imaging path to navigate to the best-expressing L2/3 FOV near the C2 barrel (~150–200-µm deep).
10. Map expression (see Procedure 3a and Fig. 5) by collecting mean two-photon images of opsin and indicator expression at their respective wavelengths (GCaMP6s: 920 nm; C1V1-Kv2.1-mRuby: 765 nm). The indicator mean image will be used as the reference image for real-time motion correction of two-photon sensory mapping data, and the opsin mean image will be used to identify neurons expressing C1V1 via manual curation.
11. For functional mapping (see Procedure 4a and Fig. 7), we need to maximize the number of sensory-responsive neurons and ensure that Suite2p has enough data to return high-quality ROIs. We will use a collection of whisker stimuli (a moving textured wall, 30-Hz dorso-ventral and rostro-caudal piezo vibration of the whole whisker pad), and we will also acquire 30 min of spontaneous activity while the animal sits on the treadmill without whisker stimulation. Wall stimuli consist of a 2-s ‘move-in’ period in which the wall moves into contact with the whiskers, a 4-s ‘in-place’ period and a 2-s ‘move-out’ period. Piezo stimuli consist of a 1-s, 30-Hz sinusoidal stimulus. All stimuli have a 10-s inter-trial interval.
12. Set up and deliver stimuli while collecting a two-photon imaging movie for each stimulus type in turn (moving wall, dorso-ventral piezo and rostro-caudal piezo; ~25 min), followed by 3 × 10-min spontaneous activity movies. Note that once each of these movies is set up in PrairieView, run *PrairieLink_RawDataStreamReg* and import the indicator mean image to trigger movie acquisition with real-time motion correction.
13. While acquiring these movies, import the opsin expression image into Naparm and manually identify all neurons expressing C1V1. Export identified C1V1 centroids (for use in Procedure 5, Step 16 below).
14. Once all functional mapping data has been acquired (Procedure 5, Steps 11 and 12), run online Suite2p by using the registered movie binary files to return ROIs and traces (~5 min per plane). Use the Suite2p curation GUI to curate ROIs (~15 min).
15. Once complete, use the resulting Suite2p output file (*proc.mat) to save images of ROIs, ROI centroids and pixel-wise local correlation.
16. Import ROI centroids and C1V1 centroids (Procedure 5, Steps 13 and 15) into Naparm. Select any centroid that appears in either of these two images but that is not too close to the edge of the FOV where photostimulation efficacy is reduced due to vignetting of the system’s photostimulation path and reduced SLM diffraction efficiency when splitting to extreme angles (if not using galvanometer hopping).
17. To perform photostimulation mapping (see Procedure 4b and Fig. 8), set up a photostimulation mapping protocol in which all potential target neurons selected in the preceding step are photostimulated with parameters similar to those that will be used in the final experiment (in our case, 10 × 25-ms spiral stimuli at 20 Hz and 500-ms duration). If the total number of targets exceeds the number that can be targeted simultaneously with sufficient photostimulation laser power per neuron (e.g., 6 mW (3 mW/µm^2^) per neuron and 600 mW total power on the sample means an upper limit of 100 neurons simultaneously), split the total number into smaller groups of equal size, each of which is small enough that all neurons within it are photostimulated with sufficient power. Note that although the total laser power on the sample should theoretically split linearly between targets, beam spots targeted to very wide angles in the FOV will be less powerful than those targeted more centrally because of constraints on the efficiency with which SLMs diffract light to wide angles^131,133^. Intensity-weighted phase masks^132,143^, which take advantage of SLMs’ ability to modulate the power in each diffracted beamlet independently, can either be used to recover equal power across targets by weighting the power in each target spot by the inverse of its distance from FOV center (and thus diffraction angle)^40,41^ or be used to equalize neuronal response magnitude by weighting each target spot’s intensity by the inverse of its target neuron’s photostimulus-evoked response magnitude such that less activatable neurons get comparatively more power than their strongly activatable counterparts^16^. See Procedure 1, Step 16 for detail. Once chosen, photostimulate each of these patterns with the stimulus parameters described above as a sequence, changing the pattern every 1 s with a sequence repetition interval of 10 s.
18. Run the photostimulation mapping protocol while acquiring two-photon imaging data with real-time motion correction to generate a Naparm movie (Procedure 4b). Add the registered binary file of this Naparm movie to the list of files used for online Suite2p analysis (i.e., along with the functional mapping data acquired previously) and re-run online Suite2p, followed by manual curation.
19. Make STA traces aligned to the onset of sensory stimuli and photostimulus epochs (Fig. 9b, Trace STAs) and analyze poststimulus periods to identify responsive neurons.
20. Find ROIs that exhibit strong sensory responses and are reliably photostimulatable (Fig. 9c–e; see the final Critical step in Procedure 4b for discussion of how to define thresholds). In this case, we selected ROIs with Δ*F*/*F* >0.3 in response to at least one sensory stimulus and Δ*F*/*F* >0.3 on ≥50% of photostimulation trials.
? TROUBLESHOOTING
21. Use the centroid locations of these ROIs as target positions for behavioral training and save associated pixel target images.
22. Import these new target images into TPBS (https://github.com/hwpdalgleish/TPBS), along with two-photon target images used for training previously (in this case, of 200 and 100 random neurons in this FOV). Set the desired stimulus power for each pixel target image and associate pixel target images to desired PyBehaviour stimulus types and variations (trial type). Set the number and rate of stimulus repetitions for each trial type and set trial-type ratios and total number of trials. If desired, set an initial trial buffer of the appropriate stimulus type (in our case, 10 trials of the easiest trial type and two-photon stimulation of 100 random neurons).
23. Once complete, generate the training protocol. This saves the list of trial types that PyBehaviour will deliver, a folder of the corresponding phase masks and the XML file and GPL file that define the stimulus order and position, respectively, in PrairieView.
24. Import the behavior and microscope configuration files into relevant software and set up a two-photon imaging movie of appropriate length in PrairieView. Ensure that PackIO is ready to record all triggers. Put the lickometer in place in front of the mouse. Check that the imaging FOV is still the same as recorded during the mapping phase: pick a bright neuron in the previously acquired mean image and ensure that the pixel location of the center of this neuron in the current FOV is the same as the previous one.
25. Begin recording with PackIO, set PrairieView Mark Points ready to receive triggers, set TPBS ready to send out triggers, begin acquiring calcium imaging movie and start the PyBehaviour session.
  ! CAUTION The total duration of functional and photostimulation mapping followed by a behavioral training session can be long (several hours). Ensure that your protocol accounts for this, i.e., that the laser powers used are not damaging the tissue over long time periods, that the objective remains sufficiently immersed, etc. If necessary, mapping and behavior can be done on different days if your microscope setup and the preparation are stable enough to be able to navigate back to the exact same FOV in terms of translation, pitch, roll and yaw. It is also essential to carefully monitor the health and behavior of experimental animals to ensure that they are not in discomfort or distress during such long periods of head restraint.
? TROUBLESHOOTING
26. Once all recordings in a given experimental animal are complete, remove the animal from the head-fixation apparatus, weigh it, record the weight and return the animal to its cage. If your behavior requires water/food deprivation, use the animal’s final weight to determine how much additional water/food to give in the cage to allow the animal to maintain a healthy weight (see your Personal Project License for requirements).
27. Analyze imaging and behavioral results in such a way as to answer your scientific question, with reference to example studies analyzing neuronal and behavioral responses to all-optical interrogation for guidance^18,20,21,37,40,41,43,44^.

## Troubleshooting

Troubleshooting advice can be found in Table 1.

## Timing

Procurement time for hardware required for an all-optical microscope: varies depending on whether one is building a system or purchasing a commercial system (e.g., from Bruker, ThorLabs or Scientifica)

Microscope setup time: varies depending on level of expertise and/or degree of collaboration with a commercial microscope manufacturer (in general, we expect this to take ~1 week; note that this is generally faster if purchasing a commercial system)

Procurement of indicator/opsin constructs: weeks if ordering a virus or using offspring from an existing transgenic line or months if setting up a new colony of transgenic mice

Procedure 1, system calibration (once the microscope/constructs have been obtained and set up): ~3 h

Procedure 2, surgery to prepare mice: ~3 h per mouse

Time between surgery and first experiment: varies. If using a full transgenic system, experiments can begin once animals have recovered from surgery (usually a few days; see your institute’s guidelines). If using AAVs to express constructs, these will usually require ~3 weeks to express sufficiently

Procedure 3a, checking indicator expression (once constructs have had the chance to express): 10–20 min per mouse

Procedure 3b, subsequent training on a behavioral task (in animals that express opsin/indicator to sufficient extents): 7–10 d per mouse (depending on the complexity of the task; more complicated tasks can take multiple weeks)

Procedure 4a, mapping functional properties of neurons that are related to your behavioral task (i.e., visual tuning in a visually guided task): 1–2 h per FOV per mouse

Procedure 4b, mapping photostimulation responses: 30 min per FOV per mouse

Procedure 5, a typical ‘final all-optical experiment’ (in which functionally tuned neurons are manipulated during a behavioral task): varies but should take ~3–5 h per FOV per mouse

Overall, for a given all-optical experiment on 1 mouse (once your system is set up and calibrated and you have decided on your opsin/indicator combination strategy: 2–4 weeks (with an additional 3 weeks prepended if relying on AAV expression)

## Anticipated results

Here, we have described the key steps involved in designing and executing a successful all-optical experiment. Efficient expression of all-optical constructs should result in hundreds of neurons in a single FOV (i.e., 500 µm × 500 µm) that are co-expressing enough opsin and indicator for photo-stimulation and readout (see Procedure 3a, Step 4 and the associated Critical step) while maintaining cell health (Fig. 5). An essential step is to rigorously test the photostimulation response of targeted neurons and factor this into the interpretation of the biological results. We describe a strategy for generating a visually intuitive map of photostimulation responses from all neurons in the desired region in as little as 30 min (Fig. 8). Using the parameters described here and assuming that expression is good and that cells are healthy, users should expect to see that >50% of neurons show a reliable, detectible photostimulation response (>0.3 Δ*F*/*F* (with GCaMP6s) on >50% of trials; see Procedure 4b, final Critical step and Fig. 9). This provides a platform for targeted activation of different numbers of neurons, in different spatial and temporal patterns, and examining the resulting effects on simultaneously measured local network activity and on behavior. Recent work has demonstrated the power of this all-optical strategy in comparing the impact of different ensembles of cells on circuit function and behavior^15,19,20,37,40–44^.

In summary, the strategy presented in this protocol, building on our efforts in different brain areas^14,36,40,41,43^ as well as those of many other groups^13,15–17,19–21,37,39,44^, represents a first attempt to provide a standardized protocol for all-optical experiments in any region of the mammalian brain, by using a range of hardware and software. Modifications of this protocol, in particular for surgical procedures tailored to particular preparations, should also allow the protocol to be applied to a wide range of species.

## Supplementary Material

Expression strategies

Reporting Summary

## Figures and Tables

**Table 1:** Troubleshooting table

Step	Problem		Possible reason	Solution
Procedure 2				
3	An excessive amount of virus leaks out of the injection site during/immediately after injection (note that a small amount of ‘backflow’ is acceptable)		Injection speed too fast and/or volume too large	Reduce injection speed and/or volume
Procedure 3a				
4	The indicator is dim		The indicator is underexpressing	Increase the concentration and/or volume of the indicator virus injected
	The indicator is very bright, does not appear to change brightness over time and/or fills nuclei		The indicator is overexpressing	Decrease the concentration and/or volume of the indicator virus injected
	The opsin reporter is dim		The opsin is underexpressing	Increase the concentration and/or volume of the opsin virus injected
	Somatically-restricted opsin expression in distal processes (the opsin is no longer somatically restricted)		The opsin is overexpressing	Decrease the concentration and/or volume of the opsin virus injected
	Neurons may have small bright punctate regions on processes in neuropil; expression in the indicator channel is generally very bright and with expression in both cytosol and nucleus		Tissue damage from needle penetration or injection	Ensure that the injection pipette is inserted slowly and carefully. Inject more slowly and/or a smaller volume. Use a thinner, sharper injection pipette
	Both opsin and indicator expressions are dim		The injection failed, the microscope was misaligned or the laser malfunctioned	Check that aliquots/stocks of the indicator/opsin constructs are viable. Avoid excessively diluting constructs and ensure that you inject a sufficient volume to see expression. During surgery, ensure that the correct volume has actually been injected (e.g., with a graduated glass pipette). If the correct volume has left the injection pipette, observe whether you see virus seeping out of the injection site during/immediately after injection. If virus is leaking out, make sure to effectively penetrate the pia (and dura if still intact) and/or try injecting more slowly and leaving the pipette in place after injection for longer Check the microscope alignment and that the laser and the collection system are functioning correctly by imaging a known good sample, such as pollen grains or a similar finely structured, highly fluorescent material
	The opsin and indicator do not co-express		Promoter or serotype conflicts	Different combinations of opsin and indicator work better in different brain regions probably due to serotype and promotor affinities; this may require some trial and error. Expressing one of the constructs transgenically, while expressing the other virally, may help. See Supplementary Table 1 for example published combinations
Procedure 4a				
6	Poor sensory-evoked responses	Weak indicator expression, suboptimal injection site, suboptimal stimulus parameters or misalignment of electrical/analysis triggers		The indicator expression may be too low to reliably report the activity evoked by the stimulus; safely increasing the level of expression, by trialing progressively increasing concentrations/volumes of virus, may help. Ensure that stimuli are presented successfully. Ensure that all frame pulses and stimulus triggers are being recorded correctly for alignment during STA. Ensure that the stimulus is appropriate for the brain region being imaged (it is beneficial to confirm area location through widefield response mapping; see Procedure 4a)
Procedure 4b				
12	Photostimulation efficacy is poor or failed completely (but expression looks good)	Optical alignment is off, or stimulus parameters are suboptimal		Check alignment of the system (including axial parfocality) and laser power on the sample (under the objective). Most issues can be found by running the spot-burning procedure on a plastic slide. If the optical calibration is correct, the issue may be that constructs are expressing well but that levels are too low for successful stimulation/readout. The stimulation parameters (power, duration and number of repeats) may be suboptimal for the preparation
Procedure 5				
20	Low number of cells that are both responsive to photostimulation and responsive to the task/sensory stimulus of interest	Expression is low; stimulus is suboptimal		The number of cells that are ultimately addressable is a product of two independent probabilities: that of being responsive to the sensory stimulus and that of being responsive to the photostimulus. Increasing opsin expression while avoiding signs of ill health will increase the numbers of photostimulatable cells. Depending on the experimental question, the definition of stimulus responsiveness could be relaxed
25	Animals lick constantly	The animal is too water restricted		Remove the animal from the experimental rig and provide it with water to allow the animal to maintain a heavier weight (>80%)
	Animal behavior is inconsistent, and/or animals give up easily when transitioning from behavior boxes to the microscope rig	Animals become frustrated or confused		Ensure that the task design and environment are kept as constant as possible, in temperature, sound proofing and white ambient noise. Keep pauses in the training session (e.g., for setting up imaging acquisitions) as short as possible

## Data Availability

The main data discussed in this protocol are available in the supporting primary research papers (refs. ^14,40,41^). The raw datasets are available for research purposes from the corresponding authors upon reasonable request.

## Box 1 Photostimulation strategies: spots versus blobs

Excitation strategies used for two-photon optogenetic stimulation of individual neurons fall into two broad categories: (i) serial excitation, which relies on galvanometer scanning (raster/spiral scanning in the image below) to scan a diffraction limited spot over the cell body, and (ii) parallel excitation, which relies on light-sculpting techniques (computer-generated holography, generalized phase contrast and temporal focusing as shown in the image below) to generate a ‘blob’ that simultaneously excites the entire cell body volume (top).

Importantly, both techniques can take advantage of combinations of fast galvanometer hopping to activate several neurons in quick succession, computer-generated holography to generate duplicate spots/blobs targeting multiple neurons simultaneously and temporal focusing to restrict the axial extent of the excitation volume. They also come with trade-offs dictating for which experiment types they will be useful. These relate to how they differ in terms of excitation volume size, laser power and time required for effective activation, as well as the mode of potential tissue damage that they risk.

Although previous theoretical work suggested that serial excitation would require a 400 times smaller excitation volume and 20 times less power than parallel excitation^93^, subsequent experimental work has demonstrated that serial excitation actually requires 2 times less power^45,92^ at the cost of time spent scanning. In practice, such differences in observed power requirements are probably affected by saturation of excitation in the focal volume, which is associated with efficient out-of-focus excitation as shown experimentally^28^. The degree to which differing photostimulation strategies (parallel versus serial) generate out-of-focus excitation has yet to be fully quantified and would be a useful direction for future research. Nonetheless, this lower requisite power means that more neurons can be targeted simultaneously by using serial excitation; however, the necessity to scan results in potentially longer and more variable delays between depolarization and action potential generation. Although both strategies have the potential to cause tissue damage, the smaller excitation volume with serial excitation, and associated greater peak power, is more likely to generate damage via nonlinear two-photon effects, whereas the larger excitation volume with parallel excitation, and higher requisite average power, is more likely to generate heating damage (although this may be outweighed by shorter excitation durations, particularly for high-density multi-site patterns; see below). The heat generated by two-photon photostimulation depends on the average power, duration, repetition rate and spatial distribution (i.e., parallel blob/spiraled spot) of each excitation site, as well as the number of excitation sites and the inter-site distance relative to the thermal diffusion length^92^. This last factor can also interact with the excitation duration of each site in a multi-site pattern such that, for sites closer than the thermal diffusion length, longer excitation of each site will result in greater heat accumulation between sites (thermal cross-talk) and thus larger overall heating effects. Therefore, for each excitation site, the average power and stimulus duration should ideally be kept as low as possible. Furthermore, for multi-site patterns, either the inter-site distance should be kept above the thermal diffusion length, or, if this is not possible, then excitation strategies favoring shorter excitation times (such as parallel illumination or fast spiral scanning) are preferable.

The choice of excitation strategy is also crucially dictated by the experimental aim and, by extension, the opsin of choice. Traditionally, opsins with slower channel kinetics, such as C1V1, were used with serial excitation methods like spiral scanning due to the necessity for current to summate throughout the scan duration to effectively drive a spike^14,15,29,30,36,40,41,43,44^. For such studies, high temporal fidelity was not a high priority because the experimental aims focused on probing the impact of the number or identity of activated neurons. Conversely, studies valuing high temporal fidelity (low latency and jitter) have tended to combine fast opsins such as Chronos^116^ or ChroME^16^ with parallel excitation methods because the simultaneous excitation of many opsin molecules with fast kinetics can rapidly and reliably drive neurons to spike. However, it should be noted that serial excitation can also generate activity with high temporal fidelity if one combines fast spiral scans (<1 ms) with very fast, sensitive opsins such as ChRmine^37^.

The figure provides a comparison of serial and parallel excitation methods, with favorable comparisons indicated in green, unfavorable in red (bottom).

In summary: serial excitation strategies are more suitable for experiments in which temporal fidelity is not a high priority, in which many neurons need to be simultaneously activated per stimulus epoch or on systems in which power is limited, particularly when multi-site stimulation patterns are sparse and/or the chosen opsin can be efficiently activated with short spirals. Conversely, parallel excitation strategies are more suited to experiments requiring exquisite temporal fidelity of action potential generation, or for dense multi-site patterns for which fast scanning is not possible. In both conditions, stimulation power, duration, repetition frequency and multi-site density must be moderated to limit tissue damage.

## Box 2 Two-photon excitation and calibration of safe and effective stimulation parameters

Two-photon excitation is a nonlinear process whereby two photons must be absorbed nearly simultaneously by a molecule for it to be excited to a higher-energy state (Fig. 10a). This occurs with a high probability only when light intensity is high, such as at the spatial focal point of a beam focused through a lens. Lasers used for two-photon absorption are typically pulsed in time to enable light intensity to be concentrated in each individual pulse, increasing the probability that two-photon excitation events occur. Apart from wavelength, such lasers are typically characterized by three key parameters central to their ability to generate two-photon excitation: time averaged power, the width of the pulses in time (pulse width) and the frequency of the pulses (repetition rate) (Fig. 10b). The probability of two-photon excitation (2PE) is proportional to:

$$2\text{PE}\propto \frac{{\text{AveragePower}}^{\text{2}}}{\text{RepetitionRate × PulseWidth}}$$

To photostimulate more cells and/or stimulate cells more strongly, one approach would be to increase the average power. However, increasing average power will lead to thermal heating of the tissue and ultimately thermal damage^91^. Another approach could be to increase the intensity of light per pulse but keep the average power constant by reducing the pulse repetition rate (Fig. 10c). Note that reducing the pulse duration could also act to increase the intensity per pulse, but this will be associated with a concomitant increase in spectral bandwidth, which may cause chromatic aberration in SLM diffraction patterns because different wavelengths will be diffracted to different angles. We note that most if not all opsin (channel and pump) photocycles are longer (on the order of tens of milliseconds^156^) than the interval between laser pulses for even sub-MHz lasers. This is very different from fluorescence imaging, for which the lifetime of the emitted fluorescence is much shorter than the interpulse interval. Opsins are thus ideally suited for use with low repetition rate, high peak power excitation (whereas fluorescence imaging necessitates high repetition rates) as we and others have found^45^ (Fig. 10d,e). A typical microscope has a power throughput of ~10% (dependent on the number of elements and the optical coatings), meaning that a typical 20-W laser will provide ~2 W of power on the sample, potentially enough to stimulate ~300 neurons at once. Typically, we stimulate no more than 100 neurons at once for a short duration to mitigate heating concerns (although this will also be influenced by the spatial distribution of target sites; see Box 1).

Although using low-repetition-rate lasers reduces the risk of thermal damage (which is proportional to average power), increasing the probability of two-photon excitation does come with an increased risk of nonlinear damage such as formation of intracellular plasma leading to ablation or lesioning of organelles^128,129^. It is therefore vital to carefully titrate the exact combination of average power, pulse width and repetition rates to be used for experiments. The activation of neurons depends on the total deposited energy, but there may be different photodamage effects associated with how that energy is deposited. We typically test a series of increasing average powers at a given laser-setting regime (wavelength, pulse width and repetition rate) and then look for successful stimulation responses as well as signs of damage such as increased baseline fluorescence (Fig. 10f–i). We then select the condition that provides successful stimulation without causing any sign of photodamage. The optimal combination of parameters will differ on the basis of the above-mentioned laser parameters (average power, pulse width and repetition rate) as well as wavelength, numerical aperture of the optical system, exposure duration and stimulus repetitions. The amount and type of non-endogenous protein expressed as well as the type of cell being tested is also likely to factor into whether the stimulated neurons respond with action potentials. Note that photodamage and photoablation (Fig. 10j) are undesirable for most experiments, but some experimental paradigms use two-photon ablation of specific neurons as a powerful manipulation^157^.

## Code availability

Custom codes used for experimental control, data acquisition, behavioral training and analysis have been deposited at Naparm (https://github.com/llerussell/Naparm), PyBehaviour (https://github.com/llerussell/PyBehaviour), 3D SLM calibration (https://github.com/llerussell/SLMTransformMaker3D), STAMovieMaker (https://github.com/llerussell/STAMovieMaker), RawDataStream (https://github.com/llerussell/Bruker_PrairieLink), Objective rotation (https://github.com/llerussell/MONPangle) and TPBS (https://github.com/hwpdalgleish/TPBS). The code in this protocol has been peer reviewed.
