# Supplementary material for: All-optical interrogation of neural circuits in behaving mice
Source: Nat Protoc. Author manuscript; Available in PMC 2024 Aug 23. (PMC7616378; doi:10.1038/s41596-022-00691-w)
Supplement: Expression strategies [file EMS198089-supplement-Expression_strategies.pdf]

nature portfolio

https://doi.org/10.1038/s41596-022-00691-w

Supplementary information

All-optical interrogation of neural circuits in behaving mice

In the format provided by the authors and unedited

Table 2: All-optical expression strategies

			Indicator							Opsin								
**Reference**	**Species and brain area**	**Co-expression strategy**	**Indicator**	**Transgenic or virus?**	**Construct or line**	**Titer (GC/ml)**	**Number of injection sites**	**Volume per injection (nL)**	**Total volume (nL)**	**Opsin**	**Transgenic or virus?**	**Construct or line**	**Titer (GC/ml)**	**Number of injection sites**	**Volume per injection (nL)**	**Total volume (nL)**	**Co-expression quantification**	**Notes**
Rickgauer et al 2014	mouse hippocampal CA1 pyramidal neurons	Transgenic + virus	GCaMP3	Transgenic	GP2.11	NA	NA	NA	NA	C1V1	Virus	AAV2/5-Camk2a-C1V1(E122T/E162T)-p2A-EYFP-WPRE	1.6 × 10^13	1	1	500-650	NR	
Szabo, Ventalon et al 2014	mouse cerebellar interneurons	Dual virus (co-suspension)	GCaMP5g	Virus	AAV2/1.hSynap.Flex.GCaMP5G.WPRE.SV40	1.47 x 10^13	1	650	650	ChR2	Virus	AAV2/1.CAGGS.flex.ChR2.tdTomato.SV40	8.4 x 10^12	1	650	650	"Cell counting gave 100% co-expression (n=513 cells from 5 mice) … 513 MLI co-expressed GCaMP5-G and ChR2- tdTomato, and no cells expressed one protein and not the other."	
Packer et al 2015	mouse barrel cortex (layer 2/3)	Dual virus (co-suspension)	GCaMP6s	Virus	AAV1-Syn-GCaMP6s-WPRE-SV40	3.22 x 10^12	1	100	100	C1V1	Virus	AAV2-CaMKIIa-C1V1(E162T)-p2A-EYFP, AAVdj-CaMKIIa-C1V1(E162T)-TS-P2A-mCherry-WPRE	NR	1	900	900	NR	Most experiments used simultaneous co-injection of opsin & indicator
Carrillo-Reid et al 2016	mouse visual cortex (layer 2/3)	Dual virus (co-suspension)	GCaMP6s	Virus	AAV1-syn-GCaMP6s-WPRE-SV40	NR	NR	NR	NR	C1V1	Virus	AAVdj-CaMKIIa- C1V1(E162T)-TS-P2A-mCherry-WPRE	NR	NR	NR	NR	"40-60% of the cells co-expressed both viruses."	
Dal Maschio, Donovan et al 2017	larval zebrafish premotor neurons	Dual transgenic	GCaMP5g, GCaMP6s	Transgenic	elavl3:GCaMP5G, UAS:GCaMP6s	NA	NA	NA	NA	ChR2(H134R)	Transgenic	UAS:ChR2(H134R)-mCherry	NA	NA	NA	200-600	NR	Zebrafish
Mardinly, Oldenburg, Pégard et al 2018	mouse barrel cortex (layer 2/3)	Transgenic + virus	GCaMP6s	Transgenic	CaMKIIa-tTA;tetO-GCaMP6s	NA	NA	NA	NA	ChroME	Virus	AAV-CAG-DIO-ST-ChroME-P2A-H2B-mRuby3	3.76-7.53 x 10^12	1	200-600	200-600	NR	See also additional details regarding co-infection with Cre or Cre transgenic as well as usage of electroporation & AAV PHP serotype
Forli et al 2018	mouse barrel cortex and visual cortex (layers 2/3 and layer 4)	Dual virus (separate injections)	jRCaMP1a	Virus	AAV1syn.NES.jRCaMP1	NR	2	200	400	ChR2(H134R)-Kv2.1	Virus	AAV1.hsyn.ChR2(H134R).EYFP.Kv2.1	NR	1	250	250	NR	
Yang et al 2018	mouse L2/3 primary visual cortex (V1)	Dual virus (co-suspension)	GCaMP6s, GCaMP6f	Virus	AAV1-syn-GCaMP6s, AAV1-syn-GCaMP6f	NR	NR	NR	NR	C1V1	Virus	AAVDJ-CaMKII-C1V1-(E162T)-TS-p2A-mCherry-WPRE	NR	NR	NR	NR		
Russell et al 2019	mouse L2/3 primary visual cortex (V1)	Transgenic + virus	GCaMP6s	Transgenic	Emx1-Cre;CaMKIIa-tTA;Ai94, CaMKIIa-tTA;tetO-GCaMP6s	NA	NA	NA	NA	C1V1-Kv2.1	Virus	AAV2/9-CaMKII-C1V1(t/t)-mRuby2-Kv2.1	2.8-8.6 x 10^13	5	150	750	NR	
Chettih and Harvey 2019	mouse L2/3 primary visual cortex (V1)	Dual virus (co-suspension)	GCaMP6s	Virus	AAV2/1-synapsin-GCaMP6s	4 x 10^12	9	40	360	C1V1-Kv2.1, ChrimsonR-Kv2.1	Virus	AAV2/9-Ef1a-ChrimsonR-mRuby2-Kv2.1, AAV2/9-Ef1a-C1V1(t/t)- mRuby2-Kv2.1	2.22 x 10^11	9	40	360	NR	Goal was sparse expression of opsin; co-injected with low titer Cre
Chen, Ronzitti et al 2019	mouse L2/3 primary visual cortex (V1)	Transgenic + virus	GCaMP6s	Transgenic	GP4.3	NA	NA	NA	NA	ReachR	Virus	AAV2/1-EF1a-ReaChR-tdTomato	NR	1	1500-2000	1500-2000	NR	
Jennings, Kim, Marshel et al 2019	mouse orbitofrontal cortex	Dual virus (co-suspension)	GCaMP6m	Virus	AAVDJ-CaMKIIα-GCaMP6m	8 x 10^12	1	500	500	bReaChES	Virus	AAV8-CaMKIIα- bReaChES-TS-p2A-mCherry	8 x 10^12	1	500	500	NR	
Marshel, Kim, Machado, Quirin et al 2019	mouse visual cortex (layer 2/3 and layer 5)	Dual virus (single construct)	GCaMP6m	Virus	AAV8-CaMKIIa-GCaMP6m-p2a-ChRmine-TS-Kv2.1-HA	4 x 10^12	2	500	1000	ChRmine	Virus	AAV8-CaMKIIa-GCaMP6m-p2a-ChRmine-TS-Kv2.1-HA	4 x 10^12	2	500	1000	NR	
Carrillo-Reid et al 2019	mouse visual cortex (layer 2/3)	Dual virus (co-suspension)	GCaMP6s	Virus	AAV1-syn-GCaMP6s-WPRE-SV40	2 x 10^13	1	400	400	C1V1	Virus	AAVdj-CaMKIIa- C1V1(E162T)-TS-P2A-mCherry-WPRE	2.7 x 10^13	1	200	200	"40-60% of the cells co-expressed both viruses."	Co-injection of opsin & indicator
Dalgleish, Russell, Packer et al 2020	mouse L2/3 primary visual cortex (V1)	Transgenic + virus<br>Dual virus (co-suspension)	GCaMP6s	Both	Emx1-Cre;CaMKIIa-tTA;Ai94, AAV1-Syn-GCaMP6s-WPRE-SV40	NA<br>NR	NA<br>1 or 5	NA<br>75 or 15	NA<br>75	C1V1,<br>C1V1-Kv2.1	Virus	AAVdj-CaMKIIa-C1V1(E162T)-TS-P2A-mCherry-WPRE, AAV2/9-CaMKII-C1V1(t/t)-mRuby2-Kv2.1, AAV2/9-CaMKII-C1V1(t/t)-mScarlet-Kv2.1	6.9 x 10^13 for Kv2.1	1 or 5	675 or 135	675	Extensive quantification in Fig 1 supplement 1	
Gill, Lerman et al 2020	mouse olfactory bulb neurons	Dual virus (co-suspension)	GCaMP6s	Virus	AAV5-Syn-GCaMP6s-WPRE-SV40	NR	1	800	800	ChrimsonR	Virus	AAV5-Syn-ChrimsonR-tdTomato or AAV1-hSyn-FLEX-ChrimsonR-tdTomato	NR	1	400	400	NR	Per hemisphere
Robinson et al 2020	mouse hippocampal CA1 pyramidal neurons	Dual virus (co-suspension)	GCaMP6f	VIrus	AAV1-Syn-GCaMP6f-WPRE-SV40	NR	1	500	500	C1V1	Virus	AAV-DJ-CaMKIIa-C1V1(E162T)-TS-p2A-mCherry-WPRE	NR	1	500	500	NR	
Daie et al 2021	mouse anterolateral motor cortex (ALM)	Transgenic + virus	GCaMP6s	Transgenic	CamKIIa-tTA;Ai94	NA	NA	NA	NA	ChrimsonR	Virus	AAV2/2 camKII-KV2.1-ChrimsonR-FusionRed	1 x 10^12	2 or 4-10	100 or 20-30	80-300	NR	
Bounds et al 2021	mouse brain	Dual transgenic	GCaMP7s	Transgenic	TITL-st-ChroME-GCaMP7s-ICL-nls-mRuby3-IRES2-tTA2; Ai203	NA	NA	NA	NA	ChroME	Transgenic	TITL-st-ChroME-GCaMP7s-ICL-nls-mRuby3-IRES2-tTA2; Ai203	NA	NA	NA	NA	~30% of neurons co-expressed both constructs	First report of a dual opsin/indicator "all-optical" transgenic mouse

All-optical co-expression strategies for in-vivo activation and imaging (i.e. inhibition and imaging not included, in vitro strategies not included, etc.) Not every combination is listed from every paper. Average taken where small ranges reported for clarity, e.g. if 1.2-1.75 ul reported, entry in table becomes 1.5

Volumes are split by indicator and opsin. In the case of co-injection of a mixed solution of viruses, the volume of each virus is listed separately. Some authors also dilute virus in PBS and/or dye for injection visualisation. Here, we report volume of virus injected, so this may not be the total volume injected into the brain; however, this table enables determining the total number of virus particles injected.

NA = not applicable

NR = not reported
