# Supplementary material for: All-optical interrogation of neural circuits in behaving mice
Source: Nat Protoc. Author manuscript; Available in PMC 2024 Aug 23. (PMC7616378; doi:10.1038/s41596-022-00691-w)
Supplement: Reporting Summary [file EMS198089-supplement-Reporting_Summary_.pdf]

nature research

Corresponding author(s): Michael Hausser, Adam Packer

Last updated by author(s): Dec 20, 2021

# Reporting Summary

Nature Research wishes to improve the reproducibility of the work that we publish. This form provides structure for consistency and transparency in reporting. For further information on Nature Research policies, see our Editorial Policies and the Editorial Policy Checklist.

## Statistics

For all statistical analyses, confirm that the following items are present in the figure legend, table legend, main text, or Methods section.

| n/a | Confirmed | |
|---|---|---|
| ☐ | ☒ | The exact sample size ($n$) for each experimental group/condition, given as a discrete number and unit of measurement |
| ☒ | ☐ | A statement on whether measurements were taken from distinct samples or whether the same sample was measured repeatedly |
| ☒ | ☐ | The statistical test(s) used AND whether they are one- or two-sided<br>*Only common tests should be described solely by name; describe more complex techniques in the Methods section.* |
| ☒ | ☐ | A description of all covariates tested |
| ☒ | ☐ | A description of any assumptions or corrections, such as tests of normality and adjustment for multiple comparisons |
| ☒ | ☐ | A full description of the statistical parameters including central tendency (e.g. means) or other basic estimates (e.g. regression coefficient) AND variation (e.g. standard deviation) or associated estimates of uncertainty (e.g. confidence intervals) |
| ☒ | ☐ | For null hypothesis testing, the test statistic (e.g. $F$, $t$, $r$) with confidence intervals, effect sizes, degrees of freedom and $P$ value noted<br>*Give P values as exact values whenever suitable.* |
| ☒ | ☐ | For Bayesian analysis, information on the choice of priors and Markov chain Monte Carlo settings |
| ☒ | ☐ | For hierarchical and complex designs, identification of the appropriate level for tests and full reporting of outcomes |
| ☒ | ☐ | Estimates of effect sizes (e.g. Cohen's $d$, Pearson's $r$), indicating how they were calculated |

*Our web collection on statistics for biologists contains articles on many of the points above.*

## Software and code

Policy information about availability of computer code

| | |
|---|---|
| Data collection | PrairieView (Bruker inc.) commercial microscope software was used to control microscopes. Blink with OverDrive Plus (MeadowLark) commercial software was used to control the SLM. PackIO (http://apacker83.github.io/PackIO/) custom software was used for data synchronisation. Naparm (https://github.com/llerussell/Naparm) custom software was used to run all-optical experiments. PyBehaviour (https://github.com/llerussell/PyBehaviour) custom software was used to run behaviour experiments. TPBS (https://github.com/hwpdalgleish/TPBS) custom software was used for two-photon all-optical behaviour experiments. SLMTransformMaker (https://github.com/llerussell/SLMTransformMaker3D) custom software was used to make microscope transforms. SLMPhaseMaskMaker3D (https://github.com/llerussell/SLMPhaseMaskMaker3D) custom software was used to make SLM phase masks. MONPangle (https://github.com/llerussell/MONPangle) custom software was used to define optimal microscope objective angle for imaging experiments. |
| Data analysis | Matlab (Mathworks Inc.) and Python were used for data analysis (using both inbuilt libraries and custom routines). STAMovieMaker (https://github.com/llerussell/STAMovieMaker) custom software was used to analyse imaging data. |

For manuscripts utilizing custom algorithms or software that are central to the research but not yet described in published literature, software must be made available to editors and reviewers. We strongly encourage code deposition in a community repository (e.g. GitHub). See the Nature Research guidelines for submitting code & software for further information.

## Data

Policy information about availability of data

All manuscripts must include a data availability statement. This statement should provide the following information, where applicable:

- Accession codes, unique identifiers, or web links for publicly available datasets
- A list of figures that have associated raw data
- A description of any restrictions on data availability

All source data used in the manuscript has been provided in previous publications. We reference these in the data availability statement in this manuscript.

# Field-specific reporting

Please select the one below that is the best fit for your research. If you are not sure, read the appropriate sections before making your selection.

☒ Life sciences ☐ Behavioural & social sciences ☐ Ecological, evolutionary & environmental sciences

For a reference copy of the document with all sections, see nature.com/documents/nr-reporting-summary-flat.pdf

# Life sciences study design

All studies must disclose on these points even when the disclosure is negative.

| | |
|---|---|
| Sample size | n/a protocol does not report any statistical tests |
| Data exclusions | n/a protocol does not report any statistical tests |
| Replication | n/a protocol does not report any statistical tests |
| Randomization | n/a protocol does not report any statistical tests |
| Blinding | n/a protocol does not report any statistical tests |

# Reporting for specific materials, systems and methods

We require information from authors about some types of materials, experimental systems and methods used in many studies. Here, indicate whether each material, system or method listed is relevant to your study. If you are not sure if a list item applies to your research, read the appropriate section before selecting a response.

## Materials & experimental systems

| n/a | Involved in the study | |
|---|---|---|
| ☒ | ☐ | Antibodies |
| ☒ | ☐ | Eukaryotic cell lines |
| ☒ | ☐ | Palaeontology and archaeology |
| ☐ | ☒ | Animals and other organisms |
| ☒ | ☐ | Human research participants |
| ☒ | ☐ | Clinical data |
| ☒ | ☐ | Dual use research of concern |

## Methods

| n/a | Involved in the study | |
|---|---|---|
| ☒ | ☐ | ChIP-seq |
| ☒ | ☐ | Flow cytometry |
| ☒ | ☐ | MRI-based neuroimaging |

## Animals and other organisms

Policy information about studies involving animals; ARRIVE guidelines recommended for reporting animal research

| | |
|---|---|
| Laboratory animals | Wild-type mice (C57/BL6), transgenic mice (Emx1-Cre, CaMKIIa-tTA, TITL-GCaMP6s, Ai94, tetO-G6s, TLX3-Cre, Grik4-Cre) |
| Wild animals | Study did not involve wild animals. |
| Field-collected samples | Study did not involve samples collected from the field. |
| Ethics oversight | All experimental procedures were carried out under Project Licence 70/14018 (PCC4A4ECE) issued by the UK Home Office in accordance with the UK Animals (Scientific Procedures) Act (1986) and were also subject to local ethical review. |

Note that full information on the approval of the study protocol must also be provided in the manuscript.
